# The effects of transcranial random noise stimulation on excitation/inhibition balance in ADHD

**DOI:** 10.1016/j.nicl.2025.103923

**Published:** 2025-12-05

**Authors:** Ornella Dakwar-Kawar, Amal Jude Ashwin Francis, Renu Arya, Noam Mairon, Jyoti Mishra, Itai Berger, Roi Cohen Kadosh, Pragathi Priyadharsini Balasubramani, Mor Nahum

**Affiliations:** a School of Occupational Therapy, Hebrew University of Jerusalem, Israel; b Department of Cognitive Science, Indian Institute of Technology Kanpur, Kanpur 208016, India; c Department of Psychiatry, University of California San Diego, USA; d Pediatric Neurology Unit, Assuta-Ashdod University Hospital and Faculty of Health Sciences, Ben-Gurion University of the Negev, Beer-Sheva, Israel; e School of Psychology, University of Surrey, Guildford, UK

**Keywords:** ADHD, Transcranial Random Noise Stimulation (tRNS), Aperiodic activity, Frontoparietal network, Default mode network, Event-related potentials, Inhibitory control

## Abstract

**Background:**

Children with attention-deficit/hyperactivity disorder (ADHD) often show aberrant neural activity, including excitation/inhibition (E/I) imbalances, atypical event-related potentials (ERPs), and neural network dysfunction. Transcranial Random Noise Stimulation (tRNS) has shown promise in modulating neural activity in ADHD.

**Methods:**

The current study examined differences in behavioral and EEG signals recorded during an inhibitory control task in children with (N = 23) and without (N = 33) ADHD. Changes in these signals were further assessed following a combined tRNS and cognitive training intervention targeting the right inferior frontal gyrus and left dorsolateral prefrontal cortex in a sham-controlled randomized trial within the ADHD group only (n = 11 and 12 for intervention and sham groups, respectively).

**Results:**

At baseline, children with ADHD showed slower reaction times, and higher commission error rates compared to healthy controls. Neurally, they exhibited elevated EEG aperiodic exponents, indicating lower E/I balance. Following intervention, we found slower task speed but fewer omission errors in the active compared to the sham group. Regression model showed reduced aperiodic exponents (β = –1.13, t(21) = –2.45, p = 0.023), decreased late ERP-P3b amplitude, and diminished theta and alpha band activity at the cortical level. There was a weaker but still significant reduction in aperiodic exponent values at follow-up compared to immediately post-treatment (β = 0.485, t(63) = 2.182, p = 0.033).

**Conclusion:**

Aperiodic exponent may serve as a useful indicator of treatment-related neural modulation and may provide complementary information to traditional ERP and event-related spectral perturbation *(*ERSP) measures, warranting further investigation in larger samples.

## Highlights

• Children with ADHD show elevated EEG aperiodic exponents vs. healthy peers.
• tRNS with cognitive training modulated neurobehavioral outcomes vs. sham in ADHD.
• tRNS reduced aperiodic exponent more consistently than ERP or ERSP indices.
• Effects of tRNS on E/I balance persisted at follow-up assessment.

## 1 Introduction

Attention-deficit/hyperactivity disorder (ADHD) is a prevalent neurodevelopmental condition characterized by inattention, hyperactivity, and impulsivity, affecting about 8 % of children and adolescents (American Psychiatric Association, 2013, Ayano et al., 2023, DuPaul et al., 2016, Greydanus et al., 2007). Children with ADHD often demonstrate pronounced deficits in executive functions (EF) particularly in attention, response inhibition and working memory (Nigg, 2001, Sadozai et al., 2024, Willcutt et al., 2005), which are thought to underlie core symptoms (Barkley 1997).

At the neural level, ADHD is associated with functional alterations in frontal and fronto-parietal networks supporting attention and cognitive control(Castellanos and Aoki, 2016, Gao et al., 2021). During inhibitory control tasks such as the Go/No-Go, children with ADHD often show reduced P300 amplitudes, an event-related potential (*ERP*) component reflecting attentional allocation and inhibition, as well as altered theta–alpha dynamics, measured through event-related spectral perturbations (ERSPs), which index (synchronization/ERS) and decreases (desynchronization/ERD), supporting cognitive control (van Bokhoven, Selten, and Nadif Kasri 2018; Kaiser et al., 2020, Michelini et al., 2022). Together, these findings indicate disrupted temporal and oscillatory dynamics in ADHD, reflecting less efficient engagement of attention and inhibitory networks. At the network level, ADHD involves disrupted coordination between large-scale brain systems such as the fronto-parietal network (FPN) and the default mode network (DMN)(Gao et al. 2019). The FPN supports sustained attention and top-down cognitive control, whereas the DMN is involved in self-referential and internally directed processes. In individuals with ADHD, the DMN often fails to deactivate during tasks, leading to attentional lapses, while the FPN shows reduced activation and connectivity, reflecting weaker cognitive control mechanisms (Castellanos and Aoki, 2016, Fateh et al., 2023, Gao et al., 2021).

Modulation of the aperiodic component of the EEG, quantified by the aperiodic exponent (AE), has also been recently suggested to characterize ADHD neural processing (van Bueren et al. 2023). The AE has been suggested as a marker of cortical excitation/inhibition (E/I) balance (Donoghue et al. 2020; Robertson et al., 2019) but see (Donoghue 2024), with higher AE indicating greater inhibitory dominance and reduced cortical excitability, whereas a lower exponent reflects increased excitation (van Bueren et al. 2023). Still, the direction of modulation of the AE in ADHD is inconsistent across studies, likely reflecting developmental, clinical, and methodological heterogeneity. Specifically, several studies have shown flatter exponents, particularly in older or medicated samples (Arnett et al., 2022a, Arnett et al., 2022b, Arnett et al., 2022c; Chen et al., 2024, Karalunas et al., 2022, Ostlund et al., 2021, Peisch and Arnett, 2024a, Pertermann et al., 2019), whereas others, including our previous findings, have demonstrated higher exponents in children with ADHD (Dakwar-Kawar et al. 2023a; Peisch and Arnett, 2024a; Robertson et al., 2019), or failed to find differences in aperiodic activity altogether (Snipes et al., 2024, Tröndle et al., 2022). Recent work further suggests that the AE indexes individual differences in neural regulation and cognitive efficiency, predicting both learning capacity (van Bueren et al. 2023) and treatment responsiveness, as flatter slopes characterize stimulant non-responders in ADHD (Anne B Arnett, Rutter, and Stein 2022c). Higher exponents may reflect a relative shift toward inhibition dominance and reduced cortical E/I balance in some ADHD populations. Here we aimed to characterize modulation in neural signals including AE in relation to ADHD status during inhibition and attention tasks.

Given that atypical E/I dynamics may contribute to ADHD symptomatology, non-invasive brain stimulation (NIBS) approaches have been explored to restore cortical balance. Studies using transcranial Direct Current Stimulation (tDCS) and transcranial Alternating Current Stimulation (tACS) in pediatric ADHD have shown mixed but encouraging behavioral and effects, including increased ERP amplitudes (P300, N200, P3a) and enhanced frontal theta activity following stimulation (Boetzel et al., 2024, Brauer et al., 2018, Brauer et al., 2021; Breitling et al. 2020; Chiang et al., 2022, Cunillera et al., 2016, Dallmer-Zerbe et al., 2020, Dubreuil-Vall et al., 2020; Westwood et al., 2022), (but see (Chiang et al. 2022). These inconsistencies likely stem from differences in stimulation duration and montage, targeted brain regions, and outcome measures, emphasizing the need for mechanistic studies using objective electrophysiological markers (Dubreuil-Vall et al. 2021).

Within this context, transcranial random noise stimulation (tRNS) has emerged as a promising technique that enhances cortical excitability through stochastic resonance, whereby weak random noise amplifies subthreshold neural activity and improves signal-to-noise processing (Terney et al., 2008). Our previous research was the first to examine the effects of tRNS combined with cognitive training in pediatric ADHD, demonstrating improvements in clinical symptoms and processing speed following intervention (Berger et al., 2021; Dakwar-Kawar et al., 2022; Nejati et al. 2024). In a follow-up study, we further found that tRNS reduces AE recorded during resting-state EEG in this population, indicating increased cortical excitability and improved neural efficiency (Dakwar-Kawar et al. 2023a). To date, however, the neurophysiological mechanisms underlying these effects remain unclear.

In the current study, we examined behavioral and neural responses during an inhibitory control task in children with and without ADHD and following combined tRNS and cognitive training. We focused on core neural markers of cognitive control, including the P3 amplitude, theta–alpha oscillatory activity at both scalp and cortical network levels (FPN and DMN), and AE derived from scalp-level EEG. We hypothesized that behavioral and neural measures would differ significantly between ADHD and control groups, and that following intervention, inhibitory performance would improve, followed by modulated E/I dynamics, aligning neural activity more closely with that of typically developing peers.

## 2 Methods

### 2.1 Study population

A sample of 56 children (age range: 6–12 years) was included: 23 children diagnosed with ADHD (20 boys), and 33 age-matched healthy comparison (HC) participants (25 boys). Recruitment period for the ADHD group was between December 2019 and December 2021 and for the HC group between 2021 and 2023. Participants with ADHD were diagnosed by a pediatric neurologist (co-author I.B.) after being referred to his clinic either by their paediatricians, general practitioners, teachers, psychologists, or parents. All participants provided verbal assent for participation and their parents provided written informed consent. All study procedures complied with the ethical standards of the relevant national and institutional committees on human experimentation and with the Helsinki Declaration of 1975, as revised in 2008. The study was approved by the Ethics Committee of the Hebrew University and by the Helsinki Committee of the Hadassah Medical Center (Jerusalem, Israel). Study design and hypotheses were pre-registered on OSF platform. The clinical trial was registered at ClinicalTrials.gov (identifier NCT03104972) and was concluded according to the pre-specified protocol with procedural changes in the trial implementation (see further details in (Dakwar-Kawar et al. 2023b)).

The full list of inclusion criteria for the ADHD group is detailed elsewhere (Berger et al. 2021; Dakwar-Kawar et al. 2023b). HC group participants were recruited as a convenience sample by word-of-mouth to friends and colleagues to match the ADHD group participants in age. Inclusion criteria for the HC group were: (a) A score below the clinical cutoff for ADHD symptoms on ADHD/DSM-5 scales; (b) Absence of attention, academic or behavioral problems based on parent reports. Participants in both groups were excluded from participation if they had a chronic neurological disease, epilepsy in participant or in first-degree relative, intellectual disability (confirmed by neurological examination) or other chronic conditions or other primary psychiatric diagnosis. Finally, we excluded girls who began the age of puberty, based on a puberty self-reported questionnaire (Petersen et al. 1988).

A power analysis using G-Power (Erdfelder and Buchner 1996) indicated that our sample size of 33 healthy controls and 23 ADHD participants allowed us to detect between-group differences with an effect size of Cohen's d = 0.4 at an alpha level of 0.05 and power of 0.8. For the treatment comparison within the ADHD group (N = 23; 11 active vs. 12 sham), our analysis was powered to detect an effect size of Cohen's d = 1.19. The sample size in the present study is small yet comparable to the sample size used in studies involving pediatric ADHD populations (Arns, Conners, and Kraemer 2013; Snyder and Hall 2006). Furthermore, for mediation analysis examining tRNS effects on symptoms and whether these effects are mediated by changes in aperiodic neural activity, a power analysis indicated that our sample of 23 participants provides 80 % power to detect mediation effects of d ≥ 1.7, appropriate for robust neurostimulation mechanisms.

### 2.2 Study design

**Fig. 1:**
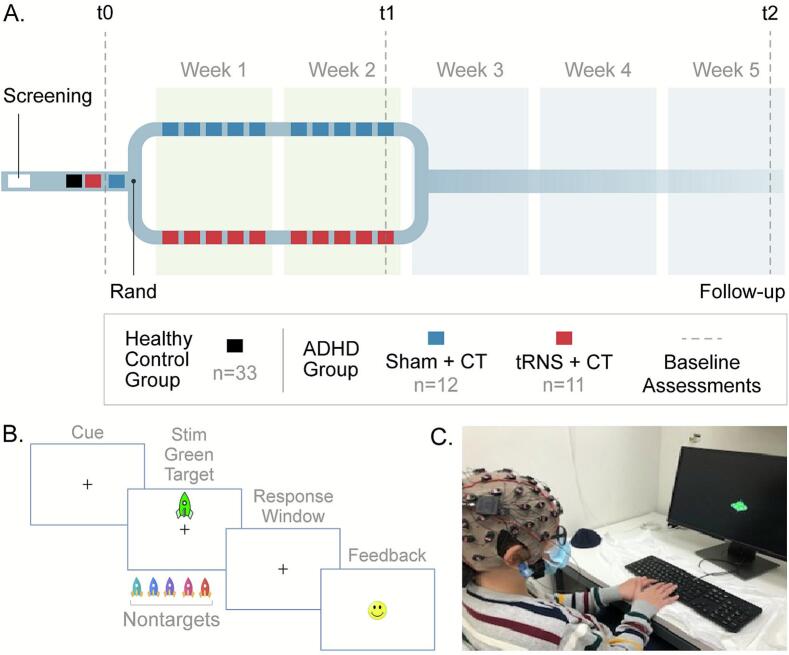
Study design. A. Following screening, eligible participants from both ADHD groups (red and blue) and the control group (black) underwent baseline assessment (t0) for clinical symptoms and completed the Go Green task while EEG was recorded. Children with ADHD were then randomized to receive 10 daily treatment sessions of either tRNS + CT or sham + CT (red and blue squares, respectively). Clinical symptom assessments and the Go Green task were repeated at the end of week 2 and again at follow-up (t1 and t2, respectively; dashed lines). B. The Go Green task. Each trial began with a fixation cue (“+”, 500 ms), followed by the presentation of a rocket stimulus (100 ms) that was either a green *Go* target or one of five *No-Go* non-target colors. Participants were instructed to respond to green rocket targets while withholding responses to distracting rockets. A response window of up to 1 s was allowed, after which feedback was displayed for 200 ms (happy/sad emoticons), followed by a 500 ms inter-trial interval (ITI). Dual happy-face emoticons provided additional positive feedback for quick, accurate responses .The task included blocks of selective attention (33% Go target trials) and response inhibition (67% Go target trials). C. Experimental setup. Example of an ERP recording session, in which EEG is recorded from children while they participate in the Go Green task. Pictures of children are included with written permission from participants and their parents.

Study design is depicted in Fig. 1. Eligible participants in both groups underwent the same baseline (t0) assessments, which included clinical symptom evaluations followed by completion of a Go/NoGo task (“Go Green”) while Event Related Potentials (ERPs) were recorded. The 23 participants in the ADHD group then completed a randomized, sham-controlled, double-blind trial: they underwent further baseline assessments, which included clinical symptom evaluation, resting-state EEG recordings, parent reports of Behavior Rating Inventory of Executive Function (BRIEF), and working memory and processing speed assessments (see (Dakwar-Kawar et al., 2023a, Dakwar-Kawar et al., 2024)). They were then randomized to receive either tRNS + CT (n = 11) or sham + CT (n = 12) for 10 sessions during a two-week period. At the end of the intervention period (t1) and at a follow up (3 weeks later; t2) they completed again the same assessment battery, including the Go Green task. Each assessment session lasted for roughly 3 h. Parents and children, as well as study assessors, were blinded to treatment assignment. All study-related activities were conducted in the Computerized Neurotherapy lab at the School of Occupational Therapy at the Hebrew University of Jerusalem.

### 2.3 Outcome measures

During each assessment session, parents completed the ADHD Rating Scale (ADHD-RS) for clinical symptom evaluations, while participants completed behavioral evaluations, resting-state EEG recordings (results reported in (Dakwar-Kawar et al., 2022, Dakwar-Kawar et al., 2023a, Dakwar-Kawar et al., 2024)), and the Go Green task with concurrent ERP recording. Intelligence was assessed using the Wechsler Intelligence Scale for Children, 4th edition (WISC-IV) (Merchán-Naranjo et al., 2012), an individually administered IQ test for children ages 6–16 years.

#### 2.3.1 Behavioural inhibitory measures − The Go Green task

This task is a modified version of the Test of Variables of Attention (Greenberg and Waldmant 1993) that measures selective attention and response inhibition (Balasubramani et al. 2021). Participants were seated at a quiet examination room at a distance of 60 cm from a computer screen (size:36 *48 cm). Colored rockets were displayed in the upper or lower central visual field across two distinct blocks. Participants were asked to respond to green rocket targets while inhibiting responses to distractors − rockets presented in five other isoluminant colors (cyan, blue, purple, pink, orange). Each trial began with a central fixation '+' (500 msec), followed by a target/distractor stimulus (100 msec), and a blank response window (up to 1 sec). Response was followed by happy or sad face emoticons (200 msec) for correct and incorrect responses, respectively, followed by a 500 msec inter-trial interval. For rapid accurate responses (100–400 msec), dual happy face emoticons provided enhanced positive feedback (Wodka et al. 2007). Each block was preceded by 4 practice trials and contained a total of 90 randomized trials, taking 5 min to complete. Performance in each block was summarized with up to 10 happy face emoticons.

The task had 2 block variations: the ‘selective attention’ block featured sparse green rocket targets (33 % of trials), while in the’inhibition’ block there were frequent green rocket targets (67 % of trials), establishing a prepotent response tendency. The requirement to suppress responses to infrequent non-targets (33 % of trials) in this block served as a measure of response inhibition (Aron 2007; Balasubramani et al. 2021; Chambers, Garavan, and Bellgrove 2009). Each participant completed an average of two blocks of selective attention and two blocks of response inhibition. The two blocks were randomly presented. The primary behavioral metric derived from this task was *commission errors*, determined by calculating the percentage of falsely pressing the button in no-go trials (also referred to as false alarms). Secondary outcomes included signal detection sensitivity (d’), calculated by subtracting the z-transformed false alarm rate from the z-transformed hit rate (i.e., correct responses to “Go” cues) (Heeger 1997). Task speed was computed as the logarithm of the inverse of the response time, log(1/RT), with RT measured in milliseconds (Barlow et al. 1980; Vandierendonck 2017). Finally, task efficiency was derived by multiplying d’ by task speed, providing an integrated measure of both accuracy and response speed (Balasubramani et al. 2021).

#### 2.3.2 Neural measures: Event related Potentials (ERPs)

ERPs recording. The full details on the EEG recording and pre-processing given elsewhere (Dakwar-Kawar et al. 2023b). In short, electrophysiological data was recorded during the Go Green task. Data was acquired using the g.Recorder system (v4.3, hereafter referred to as the research EEG system, g.Tec, Schiedlberg, Austria) connected to a g.Nautilus wireless EEG electrode cap placed on the participant's head according to the International 10–20 system (Easy Cap), using known anatomical landmarks. We used the standard 32 EEG electrode placements recordings.

ERP data analysis. EEG data were processed in EEGLAB (Delorme and Makeig 2004), with custom MATLAB scripts, including filtering (1–40 Hz), common-average referencing, and artifact removal via RSBL (Ojeda, Kreutz-Delgado, and Mullen 2018)(. Power spectra were derived with FFT for theta (4–7 Hz), alpha (8–13 Hz), and beta (13–30 Hz) bands at F3, F8, and Fz—sites relevant for aperiodic exponent changes following tRNS (Van van Bueren et al., 2022; Dakwar-Kawar et al. 2023a).We applied spectral parameterization with FOOOF (Donoghue et al. 2020)to separate periodic and aperiodic components, though only aperiodic values were analyzed here.

Channel level analysis. We examined (A) aperiodic exponents, (B) theta ERS, (C) alpha ERS, and (D) P3a/P3b components using wavelet-based ERSP and time windows of 250–350 ms and 400–600 ms, respectively. P3a was excluded when absent in a large portion of participants.

Cortical level analysis. We performed source localization (Balasubramani et al. 2021) filtered into theta, alpha, and beta bands, using an age-matched head model (Richards et al., 2016, Richards and Xie, 2015; Sanchez, Richards, and Almli 2012), Openmeeg (Gramfort et al. 2010), and RSBL (Ojeda et al. 2018) to compute source power within Desikan–Killiany atlas regions (Desikan et al. 2006). This analysis complemented the sensor-level findings by providing improved spatial specificity and allowing network-level interpretation of cortical activity. Analyses focused on the frontoparietal network (FPN) and default mode network (DMN). Full methodological details are provided in Supplementary Material 2**.**

#### 2.3.3 Exploratory outcome measures

We exploratorily analyzed pre-registered additional behavioral outcomes, including mean reaction time (RT), RT variability, hits, omission errors, and correct inhibitions across both attention and inhibition blocks. Neural exploratory outcomes included the P3 ERS in the beta frequency band of channel-level activity (as described above). These results are described fully in Supplementary Material 4**, Supplementary** Table S1.

#### 2.3.4 Study interventions

A detailed description of the study interventions is given elsewhere (Berger et al. 2021; Dakwar-Kawar et al. 2022). In short, participants completed computerized CT along with either tRNS (tRNS + CT arm) or sham (sham + CT arm) for 20 min / day for 10 days during a 2-week period. In the active condition, stimulation was delivered at 0.75  mA (peak-to-peak) using high-frequency (100–640  Hz) tRNS simultaneously over the left dorsolateral prefrontal cortex (dlPFC) and right inferior frontal gyrus (IFG), with electrodes positioned at F3 and F8 according to the International 10–20 system on the tES cap. The sham condition used the same montage as in the active tRNS, but here the 30 sec of ramp up of the current from 0 to 0.75 mA (peak-to-peak) was immediately followed by 30 sec ramp down period to 0 mA, such that participants did not receive active stimulation between ramp-up and down. This method has been shown to provide effective blindness of the stimulation condition as both active and sham tES would lead to slight itching sensation that would disappear due to scalp habitation (Sheffield et al. 2022; Greinacher et al., 2019; Gandiga et al. 2006; Nitsche and Paulus 2000; Ambrus, Paulus, and Antal 2010). Randomization and Blinding assessments as well as side effects and safety issues are mentioned in detail elsewhere (Dakwar-Kawar et al. 2023a) and are not discussed further here.

### 2.4 Statistical analyses

All statistical analyses were conducted using R. Study staff who conducted the analyses were blind to group assignment during pre-processing and analysis of all measures. Overall, there was less than 4 % missing data in the entire dataset, which stem from missing data in ERP recordings, as well as missing daily treatment sessions. This was due to movement restrictions imposed during the COVID-19 pandemic, which affected arrivals to the lab and to schools and ERP recordings that could not be analyzed due to technical issues that made some ERP recordings unusable for analysis.

Before statistical testing, outlier data, defined as values 3 SDs above or below the group mean of each measure, were removed from further analyses (Aguinis, Gottfredson, and Joo 2013). The range of outliers across variables did not exceed 2 % in the behavioural outcomes and in the ERP recordings it did not exceed 6 %. There were no significant group differences in terms of missing data, nor in outlier variables in all time points (p > 0.5).

Demographic characteristics of age, gender, and estimated IQ (Merchán-Naranjo et al., 2012) were compared using Student’s t-tests and chi-square tests for independent samples. ADHD symptoms were compared between groups using separate one-way MANOVAs, using the IBM SPSS Statistics version 25 (IBM Corp., Armonk, N.Y, USA). Linear mixed effects models (LMMs) were used to examine differences between ADHD and control groups, as well as to assess treatment effects within the ADHD group. LMMs account for within-subject correlations and for associations induced by repeated measurements. To conduct LMM analyses, we used the R-package nlme with maximized log-likelihood on the outcome measures, and subjects as the random factor. To examine treatment effects broadly on the frontal region at the channel level, we expanded our model to include electrode as a random intercept, incorporating data from both stimulated sites (F3 and F8) and the midline frontal electrode (Fz, see (Dakwar-Kawar et al. 2023a)). Additionally, to evaluate treatment effects at the neural circuit dynamics level following source localization analysis, we further extended our model by adding source as a random intercept, which encompassed data from both the FPN and DMN. Channels (F3, F8, Fz) and cortical networks (FPN, DMN) were included in the mixed-effects models with electrode/network specified as a random intercept to account for within-subject variation across electrode sites. This approach aligns with multilevel modelling practices in EEG research (Riha et al., 2020, Volpert-Esmond et al., 2021), which recommend modelling electrode-level variance rather than averaging across channels.

We examined outcomes immediately post-treatment (t1) and at a 3-week follow-up (t2) for each condition and included Treatment arm (tRNS + CT, Sham + CT) and Time (t1 and t2) as predictors. Baseline performance was added as a covariate to all models that examined treatment effects, allowing for better adjustment for minor differences in the pre-treatment means. Treatment is coded as a factor with two levels (sham and tRNS), with sham serving as the reference group. Time is also coded as a factor with two levels (t1 and t2), with post-treatment (t1) used as the reference level for modeling follow-up effects.

To ensure consistency across all primary, secondary, and exploratory outcome measures, we report results from models that included the Time × Treatment interaction term. For clarity, and to directly assess treatment effectiveness, we focus on reporting interaction effects. For all neural measures (except the aperiodic offset), the Time × Treatment interaction was significant, while no significant interaction effects were observed for the behavioral measures and for the aperiodic offset. Furthermore, model comparison analyses for the behavioral outcomes the aperiodic offset revealed that the more complex model, which included the interaction term, did not provide a better fit than a simpler model containing only the main effects of stimulation and time (ANOVA model comparison: p-values ranged from 0.548 to 0.89). As such, we report the more parsimonious model for behavioral outcomes and the aperiodic offset. Finally, when analyzing group differences at baseline (t0), we consistently used simpler models that included only the main effect of group type.

For all measures, we verified that the residuals were normally distributed using a q-q plot and the Shapiro-Wilk normality test. Neural measures were not normally distributed; we therefore applied log10 transformations to normalize these measures. Finally, we conducted a mediation analysis to examine whether the effect of stimulation (tRNS + CT vs. sham + CT) on ADHD symptom severity is mediated by changes in aperiodic neural activity, using the aperiodic exponent as the primary outcome given its central role in the proposed mechanism.

To address potential concerns related to small sample size and distributional assumptions, additional post hoc robustness checks and stability were performed using permutation (10,000 resamples) and non-parametric bootstrap analyses with replacement (2,000 iterations) on the mixed-effects models.

## 3 Results

### 3.1 Demographic characteristics of the study population

Demographic characteristics of the sample are given in Supplementary Material 1. The study CONSORT diagram is given in Fig. S1. For the HC group, 33 children were assessed for eligibility and included in the study. For the ADHD group, 25 children were assessed, 24 were randomized, and 23 successfully completed the study. One participant was excluded from the study due to difficulties complying with the required frequent arrival to the lab for treatment during the COVID-19 pandemic.

There were no significant between-group differences in age, gender, or estimated IQ (see **Supplementary** Table S2**)**, nor between the two ADHD groups in the intervention study (tRNS + CT, Sham + CT; **Supplementary** Table S3).

### 3.2 Baseline analysis: ADHD vs. HC

Analyses of all behavioral and neural outcome measures from the attention blocks are presented below. Results for the inhibition blocks are provided in full in Supplementary Material 3, with the exception of key significant results, which are detailed below.

#### 3.2.1 Behavioral results

Behavioral outcomes are summarized in Table 1. A significantly higher rate of commission errors (i.e., worse task performance; primary outcome) was found for the ADHD compared to the HC group (β = -0.62 (SE = 0.26), t(53) = -2.36, p = 0.02). Significant between-group differences were also seen for the secondary outcomes, including lower d’ (β = 0.731 (SE = 0.255), t(54) = 2.86, p = 0.006)), slower task performance (reduced task processing speed; β = 0.542 (SE = 0.264), t(54) = 2.051, p = 0.045) and reduced efficiency (the product of d’ and task speed; β = 0.799 (SE = 0.252), t(54) = 3.176, p = 0.003) in the ADHD compared to the HC group. All outcomes indicate worse mean performance for the ADHD group compared to the HC group on the attention blocks.

**Table 1:** Baseline data (ADHD vs. HC). A regression model of the primary and secondary outcome measures at baseline (t0), examining group effects (ADHD, HC) during the attention block of the ‘Go Green’ task.

	**Β**	**Std Error**	**DF**	**t value**	**P value**
**Primary outcome measures**					
**Behavioral: Commission error**					
Intercept	0.361	0.200	53	1.803	0.077
Group: HC	-0.620	0.262	53	-2.363	*0.022

**Neural measures**					
**Aperiodic exponent (frontal midline region)**					
Intercept	0.286	0.194	110	1.469	0.145
Group: HC	-0.525	0.253	54	-2.075	*0.043

**P3b: amplitude**					
Intercept	-.007	.206	95	-.03	0.97
Group: HC	.13	.27	52	.47	0.63

**ERS in the theta band frequency: Early component P3a**					
Intercept	0.096	0.212	106	0.451	0.653
Group: HC	-0.147	0.278	53	-0.527	0.601

**ERS in the theta band frequency: Late component P3b**					
Intercept	0.054	0.220	105	0.247	0.805
Group: HC	-0.006	0.287	54	-0.020	0.984

**Cortical source localization of FPN & DMN within theta frequencies**					
**Early component P3a**					
Intercept	-0.006	0.157	54	-0.039	0.969
Group: HC	0.013	0.205	54	0.065	0.948

**Cortical source localization of FPN & DMN within theta frequencies**					
**Late component P3a**					
Intercept	0.116	0.169	55	0.686	0.496
Group: HC	-0.198	0.220	54	-0.901	0.372

**Secondary outcome measures**					
**Behavioral:**					
**sensitivity (d’)**					
Intercept	-0.431	0.196	54	-2.196	*0.032
Group: HC	0.731	0.255	54	2.860	**0.006
**Task speed**					
Intercept	-0.319	0.203	54	-1.575	0.121
Group: HC	0.542	0.264	54	2.051	*0.045
**Task efficiency**					
Intercept	-0.471	0.193	54	-2.438	*0.018
Group: HC	0.799	0.252	54	3.176	**0.003

**Neural**					
**ERS in the alpha band frequency: Early component P3a**					
Intercept	.01	.02	108	.93	.35
Group: HC	-.013	.026	52	- .52	.61

**ERS in the alpha band frequency: Late component P3b**					
Intercept	.095	.21	108	.44	.66
Group: HC	- .085	.28	53	- .3	.76

**Cortical source localization of FPN & DMN within alpha frequencies**					
**Early component P3a**					
Intercept	.01	.15	55	.016	.98
Group: HC	-.002	.2	54	-.001	.992

**Cortical source localization of FPN & DMN within alpha frequencies**					
**Late component P3b**					
Intercept	.07	.15	56	.45	.65
Group: HC	-.11	.19	54	-.59	.56

#### 3.2.2 Neural Results: Channel and cortical levels

Neural outcomes are summarized in Table 1.

**Fig. 2:**
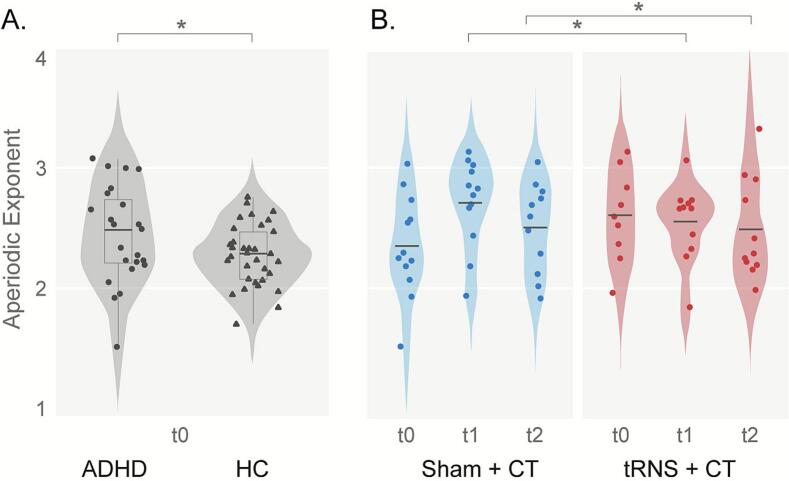
*Aperiodic Exponent values in frontal regions (F3, Fz and F8):* attention task. A*. Baseline data.* Aperiodic exponent values for participants in the ADHD (left, circles) and HC (right, triangles) groups. B. *Treatment effects*. Aperiodic exponent values at baseline (t0), post-treatment (t1) and at follow-up (t2). Values are shown for Sham + CT (left, blue) and for the tRNS + CT (right, red) groups. Horizontal lines represent mean value for each group. *p < 0.05; **p < 0.005

***Aperiodic measures: Exponent and Offset***. Significant between-group differences were seen in the attention task (β = -0.525 (SE = 0.25), t(54) = -2.075, p = . 043), indicating higher aperiodic exponent in the ADHD group compared to the HC group (Fig. 2A). No significant between-group differences were seen in the aperiodic offsets.

***Channel Level P3 components: Evoked Response Potentials (ERPs) and ERS in the theta and alpha band frequencies.*** There were no significant between-group differences in the amplitude of the P3 components at the channel level, nor at the ERS in the theta and alpha band frequencies.

***Cortical Level.*** There were no significant between-group differences at the cortical level, based on analysis of the cortical source localization of the EEG data filtered within theta and alpha frequencies in the FPN and DMN.

### 3.3 Treatment effects in the ADHD group

The full set of results describing changes in behavioral and neural signals following treatment comparing tRNS + CT vs. sham + CT in the ADHD group are summarized in Table 2. Below we present data from the attention blocks, while results for the inhibition blocks are provided in Supplementary Material 3, with the exception of key significant results, which are presented below.

**Table 2:** Treatment Data. A regression model of the primary and secondary outcome measures post-treatment (t1) and at a 3-week follow-up (t2), while covarying for baseline scores during the ‘Go Green’ task.

	**Β**	**Std Error**	**DF**	**t value**	**P value**
**Primary outcome measures**					
**Behavioral: Commission error**					
Intercept	0.618	0.526	22	1.176	0.252
Baseline score	0.521	0.198	19	2.625	0.017*
Time	0.216	0.163	19	1.332	0.199
Treatment	-0.388	0.310	19	-1.251	0.226

**Neural measures**					
**Aperiodic exponent (frontal midline region)**					
Intercept	1.124	0.320	63	3.513	**0.001
Baseline score	0.386	0.119	45	3.247	**0.002
Time	-0.641	0.157	63	-4.070	***0.000
Treatment	-1.133	0.461	21	-2.457	*0.023
Time*Treatment	0.485	0.222	63	2.182	*0.033

**P3b: amplitude**					
Intercept	0.122	0.365	51	0.335	0.739
Baseline score	0.006	0.106	42	0.059	0.953
Time	-0.033	0.171	51	-0.193	0.848
Treatment	-0.937	0.519	21	-1.806	0.085
Time*Treatment	0.547	0.234	51	2.335	0.024*

**ERS in the theta band frequency: Early component P3b**					
Intercept	-0.290	0.352	62	-0.823	0.414
Baseline score	0.128	0.146	43	0.880	0.384
Time	0.206	0.160	62	1.287	0.203
Treatment	-0.563	0.509	21	-1.106	0.281
Time*Treatment	0.475	0.229	62	2.068	*0.043

**ERS in the theta band frequency: Late component P3b**					
Intercept	-0.263	0.365	61	-0.722	0.473
Baseline score	-0.006	0.132	45	-0.042	0.967
Time	0.261	0.147	61	1.775	0.08
Treatment	-0.225	0.528	21	-0.426	0.674
Time*Treatment	0.127	0.212	61	0.599	0.551

**Cortical source localization of FPN & DMN within theta frequencies**					
**Early component P3a**					
Intercept	.17	.42	39	.41	0.69
Baseline score	44.03	54.43	20	2.88	0.009*
Time	-.55	.61	21	-.9	.38
Treatment	-0.1	0.25	39	-.41	0.69
Time*Treatment	.23	.37	39	.62	0.54

**Cortical source localization of FPN & DMN within theta frequencies**					
**Late component P3b**					
Intercept	11.63	10.42	42	1.12	0.27
Baseline score	-1.9	1.68	22	-1.13	0.27
Time	-.07	.62	21	-.11	0.91
Treatment	.13	.27	42	0.49	0.631
Time*Treatment	-.012	.38	42	-.03	0.98

**Secondary outcome measures**					
Behavioral:					
**Signal detection sensitivity d’**					
Intercept	-2.9169838	1.1949127	22	2.4411689	-0.0231
Baseline score	-2.92	1.19	22	-2.4	0.02*
Time	-0.13	.25	19	-0.5	0.62
Treatment	.18	0.29	19	.62	0.54

**Task speed**:					
Intercept	24.93	5.15	22	4.84	0.0001*
Baseline score	9.35	1.93	19	4.84	0.0001*
Time	-.03	.18	19	-.19	0.85
Treatment	.03	.26	19	.13	0.89

**Task efficiency**					
Intercept	-1.57	.54	22	-2.92	0.008*
Baseline score	1.66	.4	19	4.15	0.001*
Time	-.07	.22	19	-.32	0.75
Treatment	.28	.27	19	1.04	0.31

**Neural measures**					
**ERS in the alpha band frequency: Early component P3a**					
Intercept	-0.401	0.332	64	-1.207	0.232
Baseline score	-0.032	0.118	43	-0.267	0.791
Time	0.051	0.187	64	0.274	0.785
Treatment	-0.546	0.470	20	-1.162	0.259
Time*Treatment	0.795	0.265	64	3.006	**0.004
**ERS in the alpa band frequency: Late component P3a**					
Intercept	0.135	0.417	60	0.323	0.748
Baseline score	0.061	0.133	41	0.461	0.647
Time	-0.006	0.148	60	-0.038	0.970
Treatment	-0.735	0.616	20	-1.195	0.246
Time*Treatment	0.573	0.216	60	2.655	*0.01

**P3b: amplitude**					
Intercept	0.122	0.365	51	0.335	0.739
Baseline score	0.006	0.106	42	0.059	0.953
Time	-0.033	0.171	51	-0.193	0.848
Treatment	-0.937	0.519	21	-1.806	0.085
Time*Treatment	0.547	0.234	51	2.335	**0.024**

**Cortical source localization of FPN & DMN within alpha frequencies**					
**Early component P3a**					
Intercept	.69	.42	40	1.65	0.11
Baseline score	44.05	23.65	21	2.59	0.017
Time	-0.52	0.61	21	-.86	0.4
Treatment	-0.46	0.26	40	-1.8	0.08
Time*Treatment	0.32	0.37	40	0.85	0.4

**Cortical source localization of FPN & DMN within alpha frequencies**					
**Late component P3b**					
Intercept	0.345	0.3983	40	0.87	0.391
Baseline score	43.636	91.1741	20	4.89	0.001*
Time	-0.44	0.58	21	.78	-0.45
Treatment	-0.28	0.25	40	-1.12	0.28
Time*Treatment	0.37	0.36	40	1.0	0.3

Std = standard; DF = degrees of freedom. *p<.05; **p<0.005; ***p<0.0005

#### 3.3.1 Behavioural results

Attention task. No significant treatment effects were found for the primary outcome measure (commission errors) at post-treatment (t1) or follow-up (t2), nor in any of the secondary behavioral outcome measures.

Inhibition task. For the secondary outcomes, we found a main effect of treatment for task speed, indicating reduction in task speed of the Go trials in the tRNS + CT compared to the sham + CT group (β = -0.63 (SE = 0.28), t(19) = -2.22p = 0.039, see Supplementary Material 3).

#### 3.3.2 Neural Results: Channel level

***Aperiodic measures: Exponent and Offset***

There was a main effect of Treatment for the aperiodic exponent, indicating decreased aperiodic exponent values following tRNS + CT compared to the Sham + CT post-intervention (β = –1.13, SE = 0.46, t(21) = –2.45, p = 0.023). Furthermore, a significant Time × Treatment interaction effect was found, indicating a less pronounced yet significant decline in the tRNS + CT group at follow-up (t2) compared to immediately post-treatment (t1; β = 0.485, SE = 0.22, t(63) = 2.182, p = 0.033), (Fig. 2B). No significant effects were observed for the aperiodic offset. To further address potential concerns related to small-sample estimation, we conducted post hoc checks using permutation and non-parametric bootstrap analyses with replacement. Both methods yielded results consistent with the main mixed-effects model, confirming the stability of the direction and magnitude of the observed group-by-time effects (e.g., for the aperiodic exponent, permutation *p* = 0.046; bootstrap Δ = –0.27, 95 % CI [–0.77, 0.23]).

***Evoked Response Potential (ERP): P3***

**Fig. 3:**
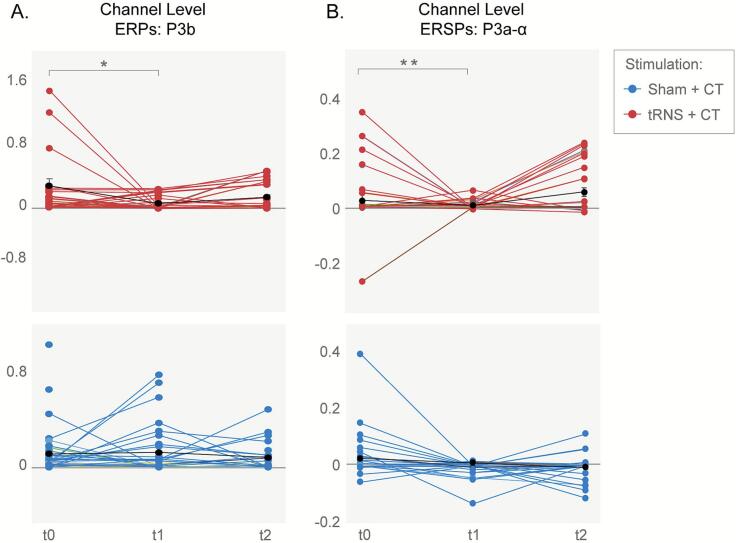
Channel Level activity during the attention task blocks: Treatment effects in ERPs and ERSPs of alpha band frequencies. (A) The P3b component of ERP amplitude for the tRNS + CT (red) and Sham + CT (blue) groups, showing changes following treatment (t1, t2) compared to baseline (t0) within each group. (B) Early (P3a) Component of the ERSP in the alpha frequency band comparing post-treatment and follow-up results to baseline within each group. Results are shown for tRNS + CT (red, top) and Sham + CT (blue, bottom) groups. Color lines represent data from individual participants; black lines show group means. *p < 0.05; **p < 0.005

There was no significant main effect of Treatment. However, a significant Time × Treatment interaction was found, indicating different post-intervention trajectories between groups (β = 0.55 (SE = 0.23), t(51) = 2.34, p = 0.024; see Fig. 3A). Specifically, the tRNS + CT group showed a reduction in ERP amplitude during the P3 window immediately post-intervention (t1), while the Sham + CT group exhibited an increase in the ERP amplitude. At follow-up (t2), the tRNS + CT group demonstrated a moderate increase (Δ = 0.06) but values did not return to baseline levels (0.13), whereas for the Sham + CT group there was a reduction in amplitude at follow-up.

***P3 components of the ERS in the theta and alpha band frequencies***

No significant main effect of Treatment was observed for either the early P3a or late P3b components of the ERS in the theta and alpha frequency bands. However, significant Time × Treatment interactions was seen for the P3a amplitude component for the theta frequencies, and in both early and late P3 components in alpha frequencies, indicating divergent post-intervention trajectories between treatment groups (theta: β = 0.48 (SE = 0.23), t(62) = 2.09, p = 0.04, early alpha: β = 0.79 (SE = 0.27), t(64) = 3.01, p = 0.003, late alpha: β = 0.57 (SE = 0.22), t(60) = 2.66, p = 0.01; respectively). Specifically, the tRNS group showed initial suppression in alpha amplitude (β = -0.94 (SE = 0.52), t(21) = -1.81, p = 0.08) followed by recovery at follow-up, while the sham group exhibited the opposite pattern showing stability (Fig. 3B).

#### 3.3.3 Neural Results: Cortical level

***EEG source localization in theta and alpha bands for FPN and DMN***.

Attention task. There were no significant main effects, nor a Time × Treatment interaction for the theta and alpha frequency bands in either early or late P3 components.

**Fig. 4:**
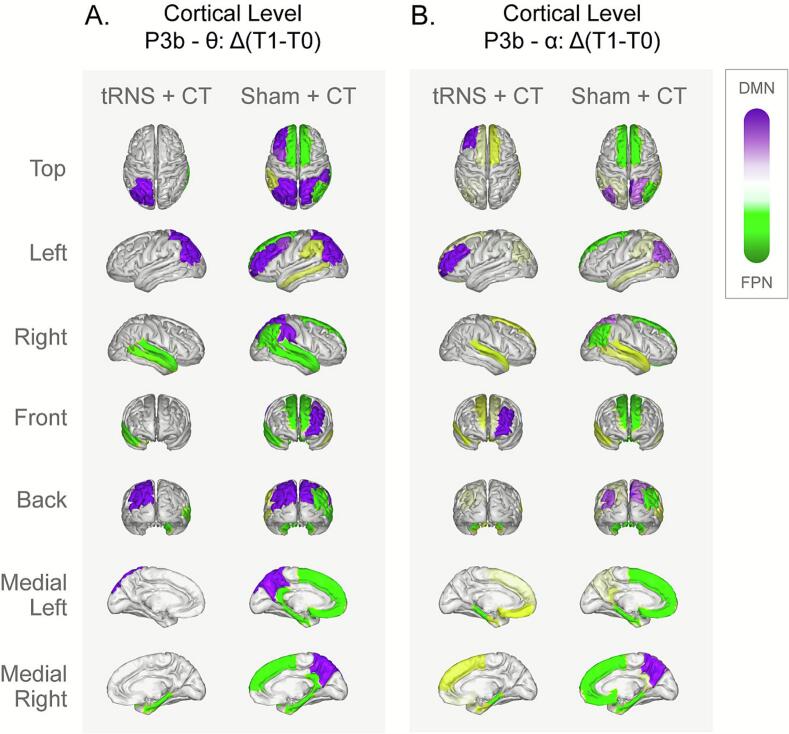
Cortical-level treatment effects during the inhibition task. Cortical source localization of EEG data filtered within the theta (A) and alpha (B) frequency in the Fronto-Parietal Network (FPN) and Default-Mode Network (DMN) for both intervention groups (left: tRNS + CT; right: Sham + CT). Δ represents network activity changes from post-treatment (t_1_) relative to baseline (t_0_). The maps illustrate relative changes in cortical activity (t_1_–t_0_) within each group: in the DMN, activity is color-coded from white → pink → purple (purple = higher activity); in the FPN, activity is color-coded from white → yellow → green (green = higher activity). Significant reductions in the late P3b component within the theta and alpha bands were observed in the FPN and DMN for the tRNS + CT group compared to the Sham + CT group. *FPN = Fronto-Parietal Network; DMN = Default-Mode Network; CT = Cognitive Training; tRNS = transcranial Random Noise Stimulation)*

Inhibition task. A significant main effect of Treatment was observed, demonstrating reduced amplitude of the late P3b component in both theta (β = -1.95, SE = 0.59, t(21) =- 3.31, p = 0.003) and alpha bands (β = -1.19, SE = 0.53, t(21) = -2.27, p = 0.034; Fig. 4) for the tRNS + CT group compared to the sham group, as identified by EEG source localization in theta and alpha bands for both FPN and DMN. These effects reflect decreased theta and alpha activity in the FPN and DMN immediately (t1) following active treatment compared to sham. Additionally, a significant Time × Treatment interaction emerged, showing that the tRNS + CT group exhibited a less decline yet significant in both theta and alpha band activity from post-treatment (t1) to follow-up (t2) compared to the sham group + CT (β = 1.15, SE = 0.34, t(41) = 3.37, p = 0.0016; β = 0.599, SE = 0.25, t(39) = 2.43, p = 0.019 for both bands, respectively).

#### 3.3.4 Exploratory analyses

Exploratory analyses assessing the intervention effects on additional behavioral measures (RTs, RT variability hits, and omission errors), as well as on ERS within the beta frequency bands are summarized in Supplementary Material 4**.** In short, no significant treatment effects were observed for the exploratory outcomes during the attention task. The only significant improvements were observed during the inhibition task, showing main effect of Treatment for hit rates and omission errors. Compared to sham, the tRNS + CT group showed increased hit rates (β = 0.88 (SE = 0.32), t(18) = 2.67, p = 0.015) and reduced omission errors (β = -0.91 (SE = 0.33), t(18) = -2.76, p = 0.013) at post- treatment (t1).

### 3.4 The association between changes in clinical, behavioral and neural outcomes following intervention

**Fig. 5:**
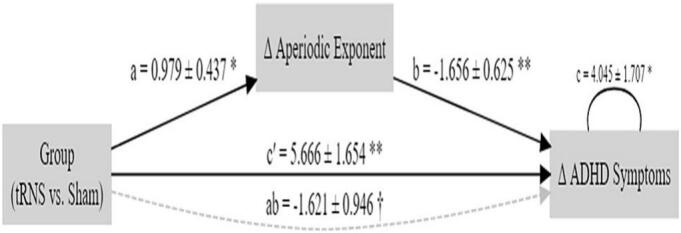
A mediation model illustrating the direct and indirect effects of treatment group (tRNS + cognitive training vs. sham + cognitive training) on change in ADHD symptoms (Δ ADHD Symptoms, calculated as post-treatment minus baseline scores [t1–t0]). The model tests whether changes in the PCA-derived component of the aperiodic exponent over frontal electrodes mediate this relationship. Path coefficients are shown for the direct effect (c′ = 5.67) and for the indirect path through the mediator (ab = –1.62), reflecting the contribution of treatment-related changes in aperiodic exponent to symptom change.

At baseline, we found higher aperiodic exponents in the ADHD group compared to the HC group. These exponents were reduced following tRNS + CT intervention. We therefore conducted a mediation analysis, examining whether changes in aperiodic activity mediate the effect of stimulation (tRNS + CT vs. sham + CT) on changes in ADHD symptom severity. Principal component analysis (PCA) was applied to the aperiodic exponents from frontal electrodes (F3, F8, and Fz) to derive a composite measure reflecting their shared activity. The results are shown in Fig. 5.

Mediation analysis revealed that group assignment (tRNS vs. Sham) significantly predicted change in the aperiodic exponent (path a: β = 0.979, SE = 0.437, 95 % CI [0.136, 1.858], p = 0.025), with the tRNS group showing greater reductions. Change in the aperiodic exponent was significantly associated with change in ADHD symptoms (path b: β = –1.656, SE = 0.625, 95 % CI [–3.079, –0.642], p = 0.008), indicating that larger reductions in the exponent predicted greater symptom improvement. The indirect effect of group on symptom change via the aperiodic exponent was negative but only marginally significant (ab: β = –1.621, SE = 0.946, 95 % CI [–3.825, –0.166], p = 0.087). The direct effect of group on symptom change, controlling for the mediator, remained significant (c′: β = 5.666, SE = 1.654, 95 % CI [2.503, 9.043], p = 0.001). The total effect of group on symptom change was also significant (c_total: β = 4.045, SE = 1.707, 95 % CI [0.762, 7.452], p = 0.018). These findings suggest that reductions in the aperiodic exponent may partially mediate the relationship between tRNS and ADHD symptom improvement.

## 4 Discussion

This study had two primary aims: (1) to examine behavioral and neural differences between children with and without ADHD during selective attention and response inhibition tasks; and (2) to evaluate immediate and longer-term effects of transcranial random noise stimulation (tRNS) combined with cognitive training (CT) over the right inferior frontal gyrus (rIFG) and left dorsolateral prefrontal cortex (lDLPFC), compared to sham stimulation.

### 4.1 ADHD status and differences in behavioral results

At baseline, children with ADHD showed impaired task performance relative to controls, with slower reaction times (RTs), higher commission error rates, and reduced signal detection sensitivity. These findings confirm prior reports of impaired inhibitory control in ADHD (Kofler et al. 2013; Lipszyc and Schachar 2010; Wright et al., 2014). Increased commission errors highlight deficits in response inhibition critical for academic and social functioning (Rubia et al. 2001). Reduced d′ sensitivity reflects difficulties distinguishing targets from distractors, suggesting impaired inhibitory control, attentional regulation, and response selection (Clark et al. 2023; Kofler et al., 2013, Kofler et al., 2013, Metin et al., 2012, Szuromi et al., 2011, Varela et al., 2024). The characteristically slower RT and increased RT variability observed here contributed to decreased processing speed but represent more than simple processing delays. In fact, they have been suggested to reflect inconsistent allocation of attentional resources that undermines the ability to engage consistently with tasks requiring sustained mental effort. Lower efficiency scores further demonstrate how these attentional deficits compound to significantly impact overall functional performance (Kofler et al., 2013; Lipszyc and Schachar 2010; Wright et al., 2014).

### 4.2 ADHD status and baseline differences in neural activity

We analyzed neural data during task performance using multiple methodologies, including more standard ERP and ERSP analyses conducted at both the channel and cortical network levels, and spectral parameterization (FOOOF) analysis performed only at the channel levels. At baseline, the only difference between groups was higher aperiodic exponents in children with ADHD, this replicates our earlier resting-state findings in the same sample (Dakwar-Kawar et al. 2023a), and suggests altered neural dynamics in ADHD, possibly reflecting disrupted E/I balance and reduced cortical efficiency (Cohen Kadosh, 2025). Similar patterns have been observed in other resting-state studies (Peisch et al., 2024b; Robertson et al., 2019), pointing to a stable neurophysiological feature across contexts.

The developmental trajectory of the AE has only recently been characterized − largely in typically developing cohorts − with a steep post-infancy decline followed by variable, sometimes U-shaped, changes across later childhood and adolescence (Donoghue, 2024, Petri et al., 2025, Stanyard et al., 2024). Against this backdrop, our results provide new evidence that elevated AE may characterize ADHD in middle childhood. This pattern may reflect delayed or altered maturation of cortical regulation, indicating that the AE captures atypical neural development in this population. Moreover, recent work links changes in AE to behavioral and clinical improvements following neuromodulation, underscoring its potential utility as a sensitive marker of treatment-related plasticity in ADHD.

Contrary to our predictions, we did not observe group differences in P3 activity, either in amplitude, oscillatory synchronization, or source localization within FPN and DMN. Prior studies often report reduced P3 amplitudes (Kaiser et al., 2020, Szuromi et al., 2011), weaker alpha suppression, and diminished theta enhancements in ADHD (Michelini et al 2022). This discrepancy can be accounted for by two factors. First, our analysis defined P3 components based on specific time windows; this is different than the standard approach often used, in which P3 components are analyzed separately for Go and No-Go trials (Balasubramani et al., 2021). The approach we took here enabled us to compare blocks with more No-Go trials (attention-demanding) to those with more Go trials (inhibitory control). This block-based analyses may capture ongoing or overlapping brain processes that span multiple trials, potentially explaining the discrepancy between our results and the typical ADHD findings of reduced P3 amplitudes and altered oscillatory activity. Second, our ADHD sample had relatively mild symptoms (Dakwar-Kawar et al., 2023a), and previous research shows stronger P3 alterations with greater symptom severity (Arnett et al. 2023). Thus, our null results may reflect both methodological differences and the clinical profile of our participants.

### 4.3 Intervention Effects: Behavioral results

The stimulation effects appeared mainly in inhibition blocks following treatment. While no significant differences were observed in speed or accuracy parameters at baseline (before treatment), children who received tRNS with CT responded more slowly on Go trials during the inhibition block but showed higher accuracy and fewer omission errors than the sham group. This pattern reflects a speed–accuracy trade-off, suggesting that the intervention promoted more deliberate responding and enhanced inhibitory control (Nejati et al. 2024).

Our results are partially consistent with the only other published study on tRNS in pediatric ADHD(Nejati et al. 2024), in which tRNS was applied over the dlPFC and vmPFC across two sessions during Go/No-Go tasks. While Nejati and colleagues reported improved No-Go accuracy with active tRNS but no effects on Go accuracy or reaction time, they observed a speed–accuracy trade-off during the Wisconsin Card Sorting Task, with longer reaction times but enhanced accuracy. In contrast, we observed a speed–accuracy trade-off within the Go/No-Go task itself, suggesting that tRNS may influence inhibitory control and processing efficiency during response inhibition paradigms. Notably, we found no changes in No-Go accuracy but observed enhanced Go performance (higher hit rates and fewer omissions). While both studies support a behavioral impact of tRNS, they differ in which performance aspects are affected, suggesting potentially distinct mechanisms, likely due to differences in stimulation sites and intervention duration. Notably, unlike our protocol, Nejati et al. applied stimulation concurrently with task performance, which may have influenced task-specific neural engagement (Nejati et al. 2024).

Importantly, this slowing-with-accuracy-gain mirrors effects seen after stimulant medication and cognitive training in ADHD(Turner et al., 2004, Warren et al., 2011). Our findings show this pattern also occurs with NIBS, which, particularly tRNS, likely enhances response inhibition by increasing excitability in cognitive control regions like the DLPFC (Chaieb et al., 2011, Estaji et al., 2024 Terney et al. 2008; Warren et al. 2011). This mechanism, consistent across various interventions, suggests response slowing may reflect more deliberate, controlled decision-making and improved executive function after NIBS (Terney et al. 2008).

### 4.4 Intervention Effects: The significance of aperiodic exponents and offsets

Our findings indicate reduced aperiodic exponents in the active compared to the sham condition post-intervention during both attention and inhibition tasks. This mirrors our earlier resting-state findings (Dakwar-Kawar et al. 2023a) and parallels results from stimulant treatment studies in ADHD (Robertson et al., 2019). However, while a recent systematic review confirms that NIBS modulates aperiodic exponents, the direction of change depends on stimulation parameters, disorder classification, and individual factors (Donoghue 2024). Further investigation into the physiological underpinnings of aperiodic activity promises deeper insights into neuropsychiatric pathophysiology (Donoghue 2024).

The reduction in aperiodic exponents observed here suggests a short-term modulation of the E/I imbalance in children with ADHD. Since elevated aperiodic exponents, as those found in our baseline data comparing ADHD and HC samples are linked to lower E/I balance and poorer behavioral performance, their decrease following stimulation may reflect a shift toward more typical neural activity patterns. These neurophysiological changes provide a mechanistic explanation for the behavioral improvements seen, such as fewer omission errors and better task performance. These preliminary findings call for further investigation of the aperiodic exponent as a potential biomarker for ADHD and treatment response in ADHD. An alternative interpretation is that tRNS-induced current fluctuations might have produced random spectral flattening rather than genuine neurophysiological modulation. However, several aspects argue against this explanation. The observed reduction in aperiodic exponent co-occurred with task- and network-specific changes in theta and alpha activity, as well as with improved inhibitory control, indicating coordinated modulation rather than random variability. Moreover, evidence from healthy populations shows that tRNS increases cortical excitability, potentially through stochastic-resonance mechanisms(van der Groen et al., 2019, Moret et al., 2019). Importantly, *van Bueren et al.* (2023) demonstrated that individuals with higher baseline aperiodic exponents, reflecting lower cortical excitability, exhibited the greatest tRNS-induced behavioral benefits, and that tRNS led to a decrease in AE independent of the baseline level. This pattern suggests that tRNS effects are state-dependent, with greater normalization occurring when baseline neural activity is suboptimal. Taken together, these findings indicate that the effects observed in the current study most likely reflect a state-dependent modulation of cortical excitability, which may be particularly pronounced in ADHD due to atypical baseline E/I dynamics. To ensure these effects were not driven by distributional assumptions given the modest sample size, we verified their consistency using permutation and bootstrap analyses, which yielded convergent results.

However, although discussion of aperiodic activity as a biomarker or as a potential marker of excitation–inhibition (E/I) balance has been increasing in recent years, emerging evidence indicates that the AE may capture broader neural regulatory processes rather than directly indexing synaptic E/I ratios. It appears to reflect a combination of neuronal synchrony, firing variability, and network-level gain control(Donoghue 2024). Moreover, developmental studies reveal nonlinear, age-dependent trajectories in AE, reflecting the gradual maturation of inhibitory control mechanisms (Petri et al. 2025) In addition, neuromodulation research shows that changes in AE following stimulation likely represent adjustments in network-level dynamics and cortical excitability rather than simple shifts in inhibition or excitation(Stanyard et al. 2024). Notably, most studies have characterized AE maturation in the resting state, leaving task-related AE modulations across the lifespan largely unexplored. Taken together, these findings highlight that while the AE is a promising neurophysiological measure, its interpretation requires careful contextualization within developmental, cognitive, and state-dependent frameworks.

### 4.5 Intervention Effects: P3 components of ERP and ERS at channel and cortical levels

tRNS produced delayed modulations in late P3b amplitude and in P3a-related theta and alpha ERS during attention and inhibition tasks. At the cortical level, these effects appeared only in inhibition blocks, with changes in the FPN and DMN, pointing to network-specific modulation of inhibitory control. The reduction in P3 amplitude may reflect adjustments in the speed–accuracy trade-off.

Although no prior work has tested ERP effects of tRNS, parallels exist with tDCS studies. For example, HD-tDCS of the rIFG has been linked to reduced P3 amplitude alongside fewer omission errors and less RT variability (Breitling-Ziegler et al., 2021; Cunillera et al. 2014), though other studies found no EEG effects (Breitling et al. 2020; Dubreuil-Vall et al. 2021; Westwood et al., 2022). At the electrode level, we observed small, non-significant P3a reductions immediately after stimulation and a different pattern at follow-up, suggesting possible compensatory adaptations. tRNS likely modulates brain oscillations by aligning endogenous rhythms to the stimulation frequency, altering excitability and synchronization (Terney et al. 2008). To date, no studies have explored tRNS or tDCS effects on ERSP during task performance. While tACS research has yielded mixed results (Boetzel et al., 2024, Brauer et al., 2018, Dallmer-Zerbe et al., 2020), this highlights a broader gap in understanding how NIBS affects early and late ERP components. Given the role of ERP markers in tracking neural impact, further research is essential.

At the cortical level, we observed neural changes exclusively during tasks requiring inhibitory control, with no changes during attention tasks. Reduced theta and alpha activity in the FPN and DMN after stimulation align with findings that repeated prefrontal tDCS can modulate connectivity in control networks (Sacca et al. 2023). This suggests multi-session tRNS may selectively strengthen neural circuits supporting inhibitory control. To further interpret these effects and disentangle their spatial and temporal characteristics, we compared the sensor- and source-level results. While sensor-level analyses captured robust group differences in scalp-recorded ERP and ERS components immediately after the intervention, source-level analyses provided greater spatial precision, revealing that the observed modulations originated primarily within frontoparietal and default-mode networks. Importantly, reduced theta and alpha activity localized to these cortical regions following tRNS + CT, and these effects persisted at follow-up, even though they were no longer evident at the scalp level. This pattern could suggest that sustained modulation was detectable only at the cortical level, likely reflecting lasting, network-specific changes that become less visible in scalp-recorded signals due to overlapping sources and volume conduction. Moreover, the source-level analysis was essential for evaluating stimulation effects within the targeted control networks (FPN and DMN), consistent with the spatial configuration of the tRNS montage. This combination of complementary analyses strengthens confidence that the reported effects reflect cortical modulation rather than transient sensor-level variability.

### 4.6 Attention vs. Inhibitory control Task: Behavioral and neural differences

The selective attention and inhibitory control blocks were intentionally designed with different Go:No-Go ratios (30:60 vs. 60:30) to manipulate cognitive demand. In the attention condition, the predominance of No-Go trials emphasized sustained vigilance and error monitoring, whereas in the inhibition condition, the frequent Go trials created a strong prepotent response tendency requiring active suppression. This design distinction has been shown to modulate both behavioral and neural responses in healthy participants, with lower Go proportions associated with improved No-Go accuracy and reduced NoGo-P3 amplitude (Zhang et al., 2024), reflecting a shift from response inhibition toward attentional control.

At the scalp level, significant effects were evident during both the attention and inhibition blocks, whereas cortical-level changes emerged primarily during inhibition. This dissociation likely reflects the distinct neural demands of the two task conditions. Although these task types emphasize different control operations, they engage overlapping large-scale networks—particularly the frontoparietal (FPN) and cingulo-opercular (CON) systems—that coordinate attentional monitoring and inhibitory regulation (Friedman and Robbins, 2022, Marek and Dosenbach, 2018). The attention block primarily recruits sustained monitoring and vigilance within these systems, whereas the inhibition block imposes greater demands on prefrontal regions responsible for executive gating and response suppression(Aron et al., 2004, Niendam et al., 2012).

Importantly, our analyses focused on FPN–DMN network dynamics, the principal targets of the tRNS montage, where no treatment-related improvement was detected at the cortical level during the attention task. Although exploratory effects were observed in other networks (e.g., auditory), these were outside the primary scope of the present study. Overall, this pattern suggests that the differences between channel- and source-level results likely reflect task-specific engagement of broader cortical systems indirectly influenced by the stimulation, rather than inconsistency in the underlying neural effects (Lai et al., 2018, Schaworonkow and Nikulin, 2022).

### 4.7 Study limitations and future directions

Although the study offers valuable insights, several limitations should be acknowledged. First, the sample size—while comparable to prior work (Looi et al. 2017; Salehinejad et al., 2020), was small, limiting statistical power and generalizability. Baseline group differences showed moderate to large effects, but treatment-related effects, though significant, occurred in a low-powered context, meaning that only large effects were detectable and null findings should be interpreted with caution.

Second, ADHD is highly heterogeneous, encompassing subtypes and comorbidities that may influence neural and behavioral responses. Despite efforts to account for this variability, the diversity in the sample limits broad generalization. Third, our assessment was restricted to short-term outcomes (three weeks post-intervention), leaving the durability of neural and behavioral changes unknown.

Finally, we targeted only the left dorsolateral prefrontal cortex and right inferior frontal gyrus. Other regions, such as the medial prefrontal cortex or anterior cingulate cortex, may also be promising targets. Future research should address these limitations by including larger, more diverse samples, incorporating long-term follow-up, and testing alternative stimulation sites to refine intervention strategies for ADHD.

## 5 Conclusions

Findings suggest that aperiodic exponent may serve as a key differentiator between children with ADHD and typically developing controls, especially in cases where traditional neural markers are absent, such as in cohorts with mild symptoms, still showing behavioral manifestations. Furthermore, the aperiodic exponent appears to be modifiable through interventions like tRNS combined with cognitive training, suggesting its potential as both a biomarker for diagnosis and a target for treatment strategies. Future research should further validate the aperiodic exponent's utility in distinguishing ADHD from typical development and explore its role in guiding effective interventions.

## 6 Declaration of generative AI and AI-assisted technologies in the manuscript preparation process

During the preparation of this work the authors used ChatGpt and Claude ai in order to improve language and readability. After using these tools, the authors reviewed and edited the content as needed and takes full responsibility for the content of the published article.

## 7 Ethical Information

All participants provided verbal assent for participation and their parents provided written informed consent. All study procedures complied with the ethical standards of the relevant national and institutional committees on human experimentation and with the Helsinki Declaration of 1975, as revised in 2008. The study was approved by the Ethics Committee of the Hebrew University and by the Helsinki Committee of the Hadassah Medical Center (Jerusalem, Israel). Study design and hypotheses were pre-registered on OSF platform. The clinical trial was registered at ClinicalTrials.gov (identifier NCT03104972) and was concluded according to the pre-specified protocol with procedural changes in the trial implementation (see further details in (Dakwar-Kawar et al., 2023a)).

## Credit authorship contribution statement

**Dakwar-Kawar Ornella:** . **Jude Ashwin Amal:** Writing – review & editing, Visualization, Validation, Methodology, Formal analysis. **Arya Renu:** . **Mairon Noam:** . **Mishra Jyoti:** . **Berger Itai:** . **Cohen Kadosh Roi:** . **Balasubramani Pragathi Priyadharsini:** . **Nahum Mor:** .

## Funding

This research was funded by a grant from the Israel Innovation Authority to Tech Innosphere Engineering Ltd. OD-K has been partially supported by Golda Meir award, granted to advanced graduate and postgraduate students in science and technology (no. 0395110) and by Zvi Yanai award, granted to advanced graduate and postgraduate Arab students (no. 3031000280); both awards of the Israeli Ministry of Science and Technology.

## Declaration of Competing Interest

The authors declare the following financial interests/personal relationships which may be considered as potential competing interests: Prof. Itai Berger serves on the advisory board of Tech InnoSphere Engineering Ltd. Prof. Roi Cohen Kadosh serves on the scientific advisory boards of Neuroelectrics Inc. and Tech InnoSphere Engineering Ltd. RCK filed a UK Patent which is manged by the University of Surrey for” method for obtaining personalized parameters for transcranial stimulation, transcranial system, method of applying transcranial stimulation”. Furthermore, Prof. Cohen Kadosh is a founder, director, and shareholder of Cognite Neurotechnology Ltd. All the other authors reported no biomedical financial interests or potential conflicts of interest..

## Data Availability

The study design and hypotheses were preregistered on the Open Science Framework (OSF). Upon publication, the de-identified data and analysis scripts will also be made openly available on the OSF platform at OSF platform.
