# Supplementary material for: The effects of transcranial random noise stimulation on excitation/inhibition balance in ADHD
Source: Neuroimage Clin. 2025 Dec 5;49:103923. doi: 10.1016/j.nicl.2025.103923 (PMC12811608; doi:10.1016/j.nicl.2025.103923)
Supplement: Supplementary Data 1 [file mmc1.docx]

## **Supplementary Material 1**

**
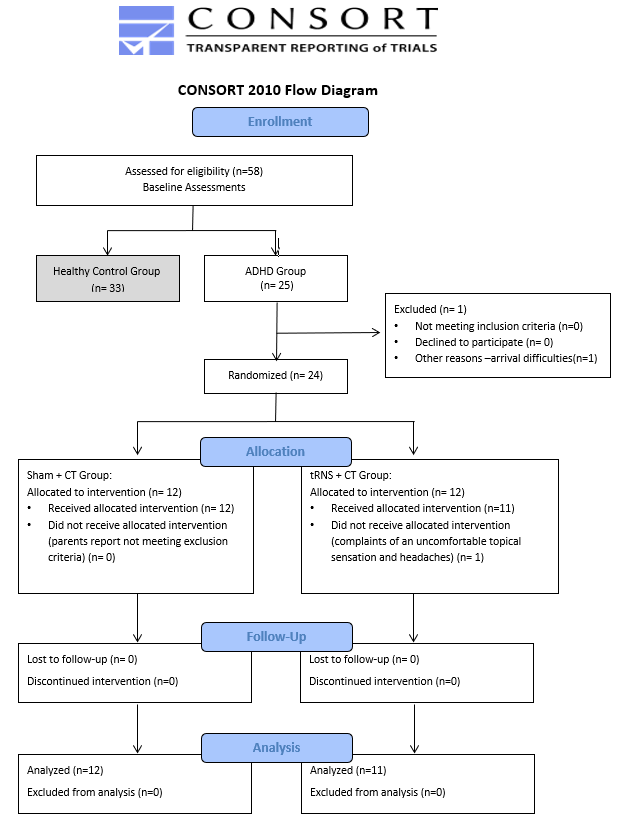
**

**Supplementary Figure S1.** **Consolidated Standards of Reporting Trials (CONSORT) flow diagram.** A total of 58 participants (25 with ADHD) were assessed for eligibility. One participant from the ADHD group was excluded due to logistical issues (arrival difficulties). The remaining participants – 24 in the ADHD group and 33 in the HC group – completed baseline assessments. Participants in the ADHD group then completed the intervention study: they were randomized into two intervention arms - tRNS combined with cognitive training (tRNS + CT) or sham stimulation combined with CT (Sham + CT). All participants in the Sham + CT arm received the allocated intervention, while one participant in the tRNS + CT arm discontinued due to discomfort and headaches. No participants were lost to follow-up. All remaining participants were included in the final analysis.

## **Supplementary Material 2. ERP Data Analysis**

ERP data analysis. Pre-processing. EEG data was analyzed using EEGLAB software (Delorme and Makeig 2004), an open-source MATLAB toolbox (freely available from <http://www.sccn.ucsd.edu/eeglab/>) and custom MATLAB scripts. Initial filtering of data between (1-40Hz) was performed, followed by common-average referencing. Although the use of a 1 Hz high-pass filter may slightly attenuate slow ERP components (Zhang, Garrett, and Luck 2024) , this approach was chosen to mitigate the substantial low-frequency noise typical of pediatric ADHD data (see Methods). Because our dataset consisted of children (6–12 yrs old) diagnosed with ADHD who exhibited abundant movement and slow baseline drifts, we applied a 1Hz high-pass filter to reduce low-frequency noise and improve signal-to-noise ratio. Prior work in children with ADHD has applied a 1Hz high-pass prior to ICA to improve classification accuracy (see Furlong et al. 2021). Additionally, methodological work on ERP filtering shows that while amplitude is attenuated by higher high-pass cut-offs (e.g., 1Hz), latency differences and relative condition comparisons are largely preserved. Given that our main comparisons rest on within‐participant or condition-difference measures (rather than absolute amplitude magnitudes), and that we verified strong correlations of peak values across subjects/conditions despite amplitude attenuation, we judged the 1Hz high-pass filter to be a reasonable trade-off between improved signal-to-noise (through movement artifact suppression) and minimal distortion of our primary effects. We specifically verified that the correlation of peak values across conditions remained high in our data, supporting that our relative comparisons are valid despite attenuation. Following filtering and referencing, we used the Recursive Spatial Bayesian Learning (RSBL) algorithm (Ojeda, Kreutz-Delgado, and Mullen 2018a) to clean the EEG data of ocular artifacts majorly and normalized to account for variations in conductance between subjects. A fast Fourier transform (FFT) was used to calculate the absolute power spectra within different specific frequency bands, focusing on theta (4-7Hz), alpha (8-13 Hz), and beta band (13-30 Hz) changes in each group. Here, we analysed data from electrodes placed over the stimulation sites (F3, F8) as well as from frontal midline area (Fz), which has shown changes in aperiodic exponent following tRNS applied to similar brain regions (Van Bueren et al. 2022; O. Dakwar-Kawar;  N. Mairon;  S.Hochman;  I. Berger; R. Cohen Kadosh; M. Nahum 2023).

We then employed a spectral parameterization approach, which enables decomposition of the neural signal into its respective periodic and aperiodic components, using FOOOF (fitting oscillations and one over *f*) (Donoghue et al. 2020). Importantly, the FOOOF tool calculates both the aperiodic value for each electrode and models the distribution features of the periodic component in the bands of interest. Given our research focus and findings from previous studies, we analyzed only the aperiodic values. interested in the aperiodic values, results for the periodic component are not reported here. AE were computed from continuous block-level EEG data rather than from single-trial ERPs. Each trial lasted approximately 1s, and the presence of strong event-related potentials can artificially increase low-frequency power, thereby inflating the 1/f slope. To ensure stable and representative estimates of the aperiodic component, artifact-free EEG segments within each task block were concatenated, and power spectra were derived using Welch’s method (1-s windows, 50% overlap). The resulting spectra were parameterized with FOOOF, and exponent and offset values were averaged per participant, condition, and frequency band of interest

Channel level analysis. (A) The *Aperiodic Exponent* was calculated based on FOOOF method. (B) *Event-Related Synchronization (ERS) in the theta band frequencies.* Because feedback stimuli (happy/sad faces) were presented after the participant’s response, the continuous EEG segments used for block-level analyses were restricted to task-related, pre-feedback activity. Feedback-locked intervals were excluded prior to spectral estimation to ensure that aperiodic and spectral measures reflected ongoing task engagement rather than outcome evaluation. Event related spectral perturbations (ERSP) were computed for frontal midline area of F3, F8 and Fz. For ERSP calculations, we performed time-frequency decomposition of the epoched data using the continuous wavelet transform (cwt) function in MATLAB’s signal processing toolbox. EEG epochs were time-locked to the presentation of each stimulus (Go or NoGo) and extended from **−**200 ms to 800 ms relative to stimulus onset. Event-related spectral perturbations (ERSPs) were computed over the same stimulus-locked epochs using wavelet decomposition. Baseline time-frequency (TF) data in the -250 msec to -200 msec time window prior to stimulus presentation were subtracted from the epoched trials (at each frequency) to observe the event-related synchronization (ERS) and event-related desynchronization (ERD) modulations (Balasubramani et al. 2021). (C) Event-Related Synchronization (ERS) in the alpha-band frequencies. (D) Early and late P3 components (a and b): The P3a amplitude was defined as the EEG mean amplitude within the 250–350 ms window, and the P3b amplitude within the 400–600 ms window . The early P3a component was not detected in 13 out of 33 participants in the healthy control group and in 11 out of 23 participants in the ADHD group; therefore, it was excluded from further analysis.

Cortical level analysis. Cortical source localization was performed on the epoched data that was filtered in theta, alpha and beta frequencies. To map the underlying neural source activations for the ERSPs, we used the block-Sparse Bayesian Learning (SBL) algorithm(Ojeda et al. 2019; Ojeda, Kreutz-Delgado, and Mullen 2018b), implemented recursively. This two-step algorithm first applies a low-resolution electromagnetic tomography (LORETA) estimate (Pascual-Marqui, Michel, and Lehmann 1995), which assumes spatial smoothness of nearby sources but can overestimate activation extent. To improve biological plausibility, the second step imposes sparsity constraints, pruning blocks of irrelevant sources and retaining only the most likely generators. Source-space activity signals were then estimated and partitioned into regions of interest (ROIs) according to the 68-region Desikan–Killiany (DK) atlas (Desikan et al. 2006a) using the Colin-27 head model (Holmes et al. 1998).

The trial averaged data of every subject was source localized for efficient approximation, and baseline corrected (Balasubramani et al. 2021). We used an age-matched head model (Richards et al. 2016; Richards and Xie 2015; Sanchez, Richards, and Almli 2012), co-registered with participant-wise EEG electrode space, and computed the lead field matrix for source localization using OpenMEEG (Gramfort et al. 2010). We computed the source power using Recursive Spatial Bayesian Learning (RSBL) algorithm (Ojeda et al. 2018a) for each network's ROI. The algorithm isolates the independent source activations in the brain by imposing the prior anatomical information and sparsity constraints, and the cortical sources are localized according to the 68-brain region Desikan-Killiany (DK) atlas (Desikan et al. 2006b). We examined the activations and co-activations mediating the brain’s dynamical network states, specifically focusing on two networks: the frontoparietal network (FPN) and the Default Mode network (DMN). FPN includes the following regions of the DK atlas:   caudalmiddlefrontal L, inferiorparietal L, parsopercularis R, precuneus L&R, rostralmiddlefrontal L, superiorparietal L&R, and supramarginal R, where R and L stand for right and left respectively. For the DMN, we included the following DK regions: superiorfrontal L&R, medialorbitofrontal L&R, isthmuscingulate L&R, supramarginal L&R, middletemporal L&R, superiortemporal R, inferiorparietal L&R, isthmuscingulate L&R, parahippocampal L&R, and entorhinal L&R.

**Supplementary Material 3. Analysis of data from inhibition blocks**

Below we present the results of the analyses of all behavioral and neural outcome measures from the inhibition blocks.

## **Baseline analysis: ADHD vs. HC**

- - 1. Behavioural Results

There were no significant between-group differences (ADHD vs. HC groups) at baseline for the primary outcome of commission errors. For the secondary outcome, efficiency scores were lower in the ADHD group compared to the controls (β=.578 (SE=.26), t(56) =2.228, p =.03). No other behavioral measures showed significant between-group differences.

- - 1. Neural Results

***Aperiodic measures: Exponent and Offset (based on FOOOF).*** There were no significant between-group differences in the aperiodic measures of exponent and offset.

***Channel Level P3 components: Evoked Response Potential (ERP) and ERS in the theta and alpha band frequencies.*** There were no significant between-group differences in the amplitude of P3 component at the channel level, nor in the ERS P3 amplitude in the theta and alpha band frequencies.

***Cortical Level.*** There were no significant between-group differences at the cortical level, based on the cortical source localization of the EEG data filtered within theta and alpha frequencies in the FPN and DMN. **a-band for FPN and DMN**

## **Treatment Effects**

Here, we compare the two intervention groups, tRNS + CT vs. sham + CT , during the inhibition blocks.

### Behavioral Results

No significant treatment effects were found on the primary outcome measure (commission errors) at the post-treatment or follow-up timepoints (t1 and t2).

### Neural Results

***Aperiodic measures: Exponent and Offset***.

The Time × Treatment interaction was non-significant for both the exponent and offset measures following treatment. For the aperiodic offset, model comparison analyses showed that the more complex interactive model did not provide a better fit than the simpler additive model with only main effects of treatment and time (ANOVA model comparison, p-values > 0.1). Consequently, we report results from the more parsimonious model for the *aperiodic offset* outcome.

During the inhibition blocks, there was a significant main effect of Treatment on the aperiodic offset, indicating lower offset values following tRNS + CT compared to Sham + CT (β = –0.8, SE = 0.27, t(21) = –2.97, p = .007). There was no significant main effect of Time, indicating that these treatment-related differences did not change significantly at follow-up (t2).

***Channel Level P3 Evoked Response Potential (ERP).***

There was a significant main effect of Treatment, indicating a decreased amplitude of the late P3b component in the tRNS + CT group compared to the Sham + CT group (β=-1.05 (SE=.48), t(21) =-2.15, p = .043). Notably, a significant Time X Treatment interaction was found, indicating less pronounced yet significant decline in the tRNS + CT group at follow-up compared to immediately post-treatment (β = 0.7, SE = 0.2, t(50) = 3.51, p = .0009).

***P3 components of the ERS in the theta and alpha band frequencies.***

No significant effects were seen in the early P3a nor in the late P3b components of the ERS in the theta band at channel level. However, for the secondary outcome (alpha band frequencies), while there was no significant main effect of Treatment, a significant Time × Treatment interaction was found for the early P3a component, indicating different post-intervention trajectories between groups (β=.67 (SE=.29), t(61)=2.28, p=.026). The estimated effect size of the treatment predictor was Cohen’s d = -0.3, 95% CI [-0.76, 0.15].

## **Supplementary Material 4. Exploratory analyses**

## **Baseline analysis: ADHD vs. HC**

- - 1. Behavioral Results

We exploratorily analyzed pre-registered additional outcome measures. Between-group differences showed the ADHD group had longer mean RTs and greater RT variability during attention blocks (mean RT: β = -.59, SE = .26, t(54) = -2.14, p = .036; RT SD: β = -.55, SE = .26, t(54) = -2.088, p = .041), and greater RT variability during inhibitory blocks (RT SD: β = .65, SE = .26, t(54) = -2.49, p = .016), though mean RT during inhibition did not differ significantly between groups (see Supplementary Table S3).

No significant between-group differences were found for hits (percentage of correct responses to Go stimuli), omission errors (missed Go responses, β = -.67, SE = .25, *t*(54) = -2.62, *p* = .011), nor for correct inhibitions to NoGo stimuli (NoGo-PC) during both attention and inhibitory blocks.

- - 1. Neural Results

There were no significant between-group differences in the channel level, nor in the P3 components of ERS in the beta band frequency.

## **Treatment Effects**

### Behavioural Results

No significant treatment effects were seen during the attention blocks on any of the exploratory behavioral measures.

### Neural Results

No significant treatment effects were observed in tasks involving selective attention and inhibitory control, as measured by the P3a amplitude component within the ERS-beta frequency range at both the channel and cortical levels.

**Supplementary Table S1. A list of exploratory behavioral and neural outcomes**

| **Measure** | **Description** |
| --- | --- |
| Behavioral measures | |
| RT mean | Mean reaction time (ms) |
| RT stdv | Standard deviation of reaction time |
| Hits | Percentage of correct responses to Go stimuli |
| Omission errors | Percentage of incorrect responses to Go stimuli |
| NoGo-PC | Percentage of correct withholding to NoGo stimuli (percentage of correct no-go) |
| Neural measures | |
| ERSPs | Event-related spectral perturbations in the beta band frequencies |
| Cortical source localization | Localization from EEG data filtered within beta frequencies |

**Supplementary Table S2.A** Baseline demographic and clinical characteristics of the ADHD and HC group participants.

| **Characteristics** | **HC** | **ADHD** |  | |  | | |  |  |  |  |
| --- | --- | --- | --- | --- | --- | --- | --- | --- | --- | --- | --- |
|  | **n=33**  **mean (SD)** | **(n=23)**  **mean (SD)** | | ***t*/χ2/ F**  **(dfh, dfe)** | | | **P Value** | | **Effect Size**  **(partial** η^2^**)** | |  |
| Age (y) | 9.42 (1.6) | 8.96 (1.4) | | 1.12 | | | .26 | |  | |  |
| Male (*n*, %) | 25, 76% | 20, 87% | | 1.6 | | | .32 | |  | |  |
| **WISC** |  |  | |  | | |  | |  | |  |
| Estimated IQ | 107.42 (23.95) | 97.39 (18.76) | | 1.68 | | | .142 | |  | |  |
|  |  |  | |  | | |  | |  | |  |
|  |  |  | |  | | |  | |  | |  |
| **ADHD symptoms (ADHD-RS)** | Wilk’s Λ =0.23 | | 100.37  (1,52) | | | **.0001***** | | | | .797 | |
| Total score | 1.4 | 11.62 | |  | | |  | |  | |  |
| Inattentiveness | .73 (.27) | 6.81 (.34) | | 199.44 (1,52) | | | **.0001***** | | .793 | |  |
| Hyperactivity-Impulsivity | .67 (.31) | 4.81 (.4) | | 67.95 (1,52) | | | .**0001***** | | .566 | |  |

**Supplementary Table S2.B** Baseline Demographic and Clinical Characteristics of tRNS+ CT and Sham + CT groups. Reproduced with permission from Dakwar-Kawar et al. (2023).

| **Characteristics** | **ADHD** | |  |  |  |
| --- | --- | --- | --- | --- | --- |
|  | **tRNS + CT (n=11)**  **mean (SD)** | **Sham + CT (n=12)**  **mean (SD)** | ***t*/χ2/ F**  **(dfh, dfe)** | **P Value** | **Effect Size**  **(partial** η^2^**)** |
| Age (y) | 9.25 (1.42) | 8.64 (1.43) | 1.13 | .30 |  |
| Male (*n*, %) | 10, 90.9% | 10, 83.3% | .29 | .59 |  |
| **WISC** |  |  |  |  |  |
| Estimated IQ | 104.5 (10.76) | 90.8 (21.2) | 2.05 | .067 |  |
|  |  |  |  |  |  |
|  |  |  |  |  |  |
| **ADHD symptoms (ADHD-RS)** | Wilk’s Λ = .99 | | .009  (1,21) | .99 | .001 |
| Total score | 10.91(1.12) | 11.08(1.47) |  |  |  |
| Inattentiveness | 6.55 (.49) | 6.67 (.74) | .02  (1,21) | .89 | .001 |
| Hyperactivity-Impulsivity | 4.36 (.85) | 4.42 (.83) | .002  (1,21) | .97 | .0001 |

**Supplementary Table S3.** A regression model was used to examine group differences (ADHD vs. HC) in exploratory outcome measures at **(A)** baseline ( t0) during the attention and inhibition blocks of the ‘Go Green’ task. Additional analyses were conducted on the same measures at **(B)** post-treatment (t1) and at a 3-week follow-up (t2).

1. **Baseline (t0)**

|  | **Β** | | **Std Error** | | **DF** | | **t value** | **P value** | |  |
| --- | --- | --- | --- | --- | --- | --- | --- | --- | --- | --- |
| **Primary outcome measures** | | | | | | | | | |  |
| **Behavioral:** **Commission error** | | | | | | | | | |  |
| Intercept | 0.201 | 0.208 | | | 56 | | 0.969 | 0.337 | |  |
| Group: HC | -0.333 | 0.267 | | | 56 | | -1.247 | 0.218 | |  |
| **Neural measures** | | | | | | | | | |  |
| **Aperiodic exponent (frontal midline region)** | | | | | | | | | |  |
| Intercept | 0.245 | 0.190 | | | 108 | | 1.292 | 0.199 | |  |
| Group: HC | -0.428 | 0.251 | | | 52 | | -1.706 | 0.094 | |  |
| \| **P3b: amplitude** \| \|  \|  \| \| --- \| --- \| --- \| --- \| \| Intercept \| -0.009 \| 0.262 \| 107 \| -0.035 \| 0.973 \| \| Group: HC \| 0.191 \| 0.340 \| 55 \| 0.562 \| 0.576 \|   **ERS in the theta band frequency: Early component P3a** | | | | | | | | | |  |
| Intercept | -0.134 | 0.198 | | | 109 | | -0.674 | 0.502 | |  |
| Group: HC | 0.289 | 0.258 | | | 54 | | 1.121 | 0.267 | |  |
| **ERS in the theta band frequency:** **Late component P3b** | | | | | | | | | |  |
| Intercept | -0.174 | 0.202 | | 108 | | -0.858 | | 0.393 |  |  |
| Group: HC | 0.370 | 0.263 | | 54 | | 1.407 | | 0.165 |  |  |
| **Cortical source localization of FPN & DMN within theta frequencies**  **Early component P3a** | | | | | | | | | |  |
| Intercept | -0.170 | 0.173 | | | 56 | | -0.981 | 0.331 | |  |
| Group: HC | 0.289 | 0.224 | | | 55 | | 1.289 | 0.203 | |  |
| **Cortical source localization of FPN & DMN within theta frequencies**  **Late component P3a** | | | | | | | | | |  |
| Intercept | -0.122 | 0.172 | | | 56 | | -0.706 | 0.483 | |  |
| Group: HC | 0.204 | 0.224 | | | 55 | | 0.910 | 0.367 | |  |
| **Secondary outcome measures** | | | | | | | | | |  |
| **Behavioral:** | | | | | | | | | |  |
| **sensitivity (d’)** | | | | | | | | | |  |
| Intercept | -0.296 | 0.204 | | | 56 | | -1.450 | 0.153 | |  |
| Group: HC | 0.491 | 0.263 | | | 56 | | 1.867 | 0.067 | |  |
| **Task speed** | | | | | | | | | |  |
| Intercept | -0.220 | 0.207 | | | 56 | | -1.064 | 0.292 | |  |
| Group: HC | 0.365 | 0.266 | | | 56 | | 1.370 | 0.176 | |  |
| **Task efficiency** | | | | | | | | | |  |
| Intercept | -0.349 | 0.202 | | | 56 | | -1.731 | 0.089 | |  |
| Group: HC | 0.578 | 0.260 | | | 56 | | 2.228 | **0.030*** | |  |
| **Neural**  **ERS in the alpha band frequency: Early component P3a** | | | | | | | | | |  |
| Intercept | -0.131 | 0.307 | | | 108 | | -0.426 | 0.671 | |  |
| Group: HC | 0.454 | 0.394 | | | 54 | | 1.153 | 0.254 | |  |
| **ERS in the alpha band frequency: Late component P3b** | | | | |  | |  |  | |  |
| Intercept | 0.061 | 0.038 | | | 108 | | 1.590 | 0.115 | |  |
| Group: HC | 0.025 | 0.050 | | | 54 | | 0.493 | 0.624 | |  |
| **Cortical source localization of FPN & DMN within alpha frequencies**  **Early component P3a** | | | | | | | | | |  |
| Intercept | -0.161 | 0.156 | | | 56 | | -1.031 | 0.307 | |  |
| Group: HC | 0.278 | 0.203 | | | 55 | | 1.369 | 0.177 | |  |
| **Cortical source localization of FPN & DMN within alpha frequencies**  **Late component P3b** | | | | | | | | | |  |
| Intercept | 0.124 | 0.169 | | | 33 | | 0.735 | 0.467 | |  |
| Group: HC | -0.328 | 0.178 | | | 22 | | -1.848 | 0.078 | |  |

**B. Post-treatment (t1) and at a 3-week follow-up (t2).**

|  | **Β** | | **Std Error** | **DF** | **t value** | **P value** |  |  |  |  |  |  |  |  |  |  |  |  |
| --- | --- | --- | --- | --- | --- | --- | --- | --- | --- | --- | --- | --- | --- | --- | --- | --- | --- | --- |
| **Primary outcome measures** | | | | | | |  |  |  |  |  |  |  |  |  |  |  |  |
| **Behavioral: Commission error** | | | | | | |  |  |  |  |  |  |  |  |  |  |  |  |
| Intercept | 1.756 | 0.761 | | 22 | 2.306 | **0.031*** |  |  |  |  |  |  |  |  |  |  |  |  |
| Baseline score | 0.266 | 0.233 | | 19 | 1.142 | 0.268 |  |  |  |  |  |  |  |  |  |  |  |  |
| Time | 0.012 | 0.220 | | 19 | 0.053 | 0.958 |  |  |  |  |  |  |  |  |  |  |  |  |
| Treatment | -0.107 | 0.348 | | 19 | -0.308 | 0.761 |  |  |  |  |  |  |  |  |  |  |  |  |
| **Neural measures** | | | | | | |  |  | | |  | | |  | |  | |  |
| Aperiodic exponent (frontal midline region) | | | | | | |  |  |  |  |  |  |  |  |  |  |  |  |
| Intercept | -0.426 | 0.326 | | 64 | -1.307 | 0.196 |  |  |  |  |  |  |  |  |  |  |  |  |
| Baseline score | 0.099 | 0.126 | | 45 | 0.784 | 0.437 |  |  |  |  |  |  |  |  |  |  |  |  |
| Time | 0.453 | 0.172 | | 64 | 2.635 | 0.011 |  |  |  |  |  |  |  |  |  |  |  |  |
| Treatment | -0.102 | 0.472 | | 21 | -0.216 | 0.831 |  |  |  |  |  |  |  |  |  |  |  |  |
| Time*Treatment | -0.296 | 0.244 | | 64 | -1.213 | 0.230 |  |  |  |  |  |  |  |  |  |  |  |  |
| **ERS in the theta band frequency: Early component P3a** | | | | | | |  | | |  | | |  | |  | |  | |
| Intercept | 0.479 | 0.439 | | 40 | 1.091 | 0.282 |  |  |  |  |  |  |  |  |  |  |  |  |
| Baseline score | -0.102 | 0.124 | | 20 | -0.822 | 0.421 |  |  |  |  |  |  |  |  |  |  |  |  |
| Time | -0.263 | 0.258 | | 40 | -1.022 | 0.313 |  |  |  |  |  |  |  |  |  |  |  |  |
| Treatment | -0.679 | 0.629 | | 21 | -1.079 | 0.293 |  |  |  |  |  |  |  |  |  |  |  |  |
| Time*Treatment | 0.340 | 0.368 | | 40 | 0.924 | 0.361 |  |  |  |  |  |  |  |  |  |  |  |  |
| **ERS in the theta band frequency: Late component P3b** | | | | | | |  | | |  | | |  | |  | |  | |
| Intercept | 1.069 | 0.414 | | 41 | 2.582 | 0.014 |  |  |  |  |  |  |  |  |  |  |  |  |
| Baseline score | 0.153 | 0.117 | | 21 | 1.316 | 0.202 |  |  |  |  |  |  |  |  |  |  |  |  |
| Time | -0.619 | 0.242 | | 41 | -2.556 | **0.014*** |  |  |  |  |  |  |  |  |  |  |  |  |
| Treatment | -1.945 | 0.588 | | 21 | -3.311 | **0.003**** |  |  |  |  |  |  |  |  |  |  |  |  |
| Time*Treatment | 1.152 | 0.342 | | 41 | 3.374 | **0.002**** |  |  |  |  |  |  |  |  |  |  |  |  |
| **Cortical source localization of FPN & DMN within theta frequencies**  **Early component P3a** | | | | | | |  |  |  |  |  |  |  |  |  |  |  |  |
| Intercept | 0.479 | 0.439 | | 40 | 1.091 | 0.282 |  |  |  |  |  |  |  |  |  |  |  |  |
| Baseline score | -0.102 | 0.124 | | 20 | -0.822 | 0.421 |  |  |  |  |  |  |  |  |  |  |  |  |
| Time | -0.263 | 0.258 | | 40 | -1.022 | 0.313 |  |  |  |  |  |  |  |  |  |  |  |  |
| Treatment | -0.679 | 0.629 | | 21 | -1.079 | 0.293 |  |  |  |  |  |  |  |  |  |  |  |  |
| Time*Treatment | 0.340 | 0.368 | | 40 | 0.924 | 0.361 |  |  |  |  |  |  |  |  |  |  |  |  |
| **Cortical source localization of FPN & DMN within theta frequencies**  **Late component P3b** | | | | | | |  |  |  |  |  |  |  |  |  |  |  |  |
| Intercept | 1.069 | 0.414 | | 41 | 2.582 | **0.014*** |  |  |  |  |  |  |  |  |  |  |  |  |
| Baseline score | 0.153 | 0.117 | | 21 | 1.316 | 0.202 |  |  |  |  |  |  |  |  |  |  |  |  |
| Time | -0.619 | 0.242 | | 41 | -2.556 | **0.014*** |  |  |  |  |  |  |  |  |  |  |  |  |
| Treatment | -1.945 | 0.588 | | 21 | -3.311 | **0.003**** |  |  |  |  |  |  |  |  |  |  |  |  |
| Time*Treatment | 1.152 | 0.342 | | 41 | 3.374 | **0.002**** |  |  |  |  |  |  |  |  |  |  |  |  |
| **Secondary outcome measures** | | | | | | |  |  |  |  |  |  |  |  |  |  |  |  |
| Behavioral: |  |  | |  |  |  |  |  |  |  |  |  |  |  |  |  |  |  |
| **Signal detection sensitivity d’** | |  | |  |  |  |  |  |  |  |  |  |  |  |  |  |  |  |
| Intercept | 1.548 | 0.574 | | 22 | 2.695 | 0.013 |  |  |  |  |  |  |  |  |  |  |  |  |
| Baseline score | 0.461 | 0.195 | | 19 | 2.371 | 0.029 |  |  |  |  |  |  |  |  |  |  |  |  |
| Time | -0.03 | 0.178 | | 19 | -0.171 | 0.866 |  |  |  |  |  |  |  |  |  |  |  |  |
| Treatment | 0.365 | 0.258 | | 19 | 1.412 | 0.174 |  |  |  |  |  |  |  |  |  |  |  |  |
| **Task speed**: |  |  | |  |  |  |  |  |  |  |  |  |  |  |  |  |  |  |
| Intercept | 0.186 | 0.267 | | 22 | 0.696 | 0.494 |  |  |  |  |  |  |  |  |  |  |  |  |
| Baseline score | 0.599 | 0.164 | | 19 | 3.660 | **0.002**** |  |  |  |  |  |  |  |  |  |  |  |  |
| Time | 0.065 | 0.109 | | 19 | 0.598 | 0.557 |  |  |  |  |  |  |  |  |  |  |  |  |
| Treatment | -0.625 | 0.281 | | 19 | -2.222 | **0.039*** |  |  |  |  |  |  |  |  |  |  |  |  |
| **Task efficiency** |  |  | |  |  |  |  |  |  |  |  |  |  |  |  |  |  |  |
| Intercept | 0.336 | 0.216 | | 22 | 1.560 | 0.133 |  |  |  |  |  |  |  |  |  |  |  |  |
| Baseline score | 0.683 | 0.177 | | 19 | 3.858 | 0.001 |  |  |  |  |  |  |  |  |  |  |  |  |
| Time | 0.014 | 0.077 | | 19 | 0.184 | 0.856 |  |  |  |  |  |  |  |  |  |  |  |  |
| Treatment | 0.158 | 0.107 | | 19 | 1.487 | 0.153 |  |  |  |  |  |  |  |  |  |  |  |  |
| **Neural measures** | | | | | | |  | |  | | |  |  |  |  |  |  |  |
| **ERS in the alpha band frequency: Early component P3a** | | | | | | |  |  |  |  |  |  |  |  |  |  |  |  |
| Intercept | 0.183 | 0.424 | | 39 | 0.431 | 0.669 |  |  |  |  |  |  |  |  |  |  |  |  |
| Baseline score | 0.063 | 0.117 | | 21 | 0.536 | 0.597 |  |  |  |  |  |  |  |  |  |  |  |  |
| Time | -0.394 | 0.616 | | 21 | -0.639 | 0.530 |  |  |  |  |  |  |  |  |  |  |  |  |
| Treatment | -0.094 | 0.236 | | 39 | -0.398 | 0.693 |  |  |  |  |  |  |  |  |  |  |  |  |
| Time*Treatment | 0.286 | 0.339 | | 39 | 0.842 | 0.405 |  |  |  |  |  |  |  |  |  |  |  |  |
| **ERS in the alpa band frequency: Late component P3a** | | | | | | |  |  |  |  |  |  |  |  |  |  |  |  |
| Intercept | 0.566 | 0.360 | | 39 | 1.570 | 0.124 |  |  |  |  |  |  |  |  |  |  |  |  |
| Baseline score | 0.264 | 0.143 | | 19 | 1.843 | 0.081 |  |  |  |  |  |  |  |  |  |  |  |  |
| Time | -0.238 | 0.172 | | 39 | -1.382 | 0.175 |  |  |  |  |  |  |  |  |  |  |  |  |
| Treatment | -1.193 | 0.527 | | 21 | -2.265 | **0.034*** |  |  |  |  |  |  |  |  |  |  |  |  |
| Time*Treatment | 0.599 | 0.246 | | 39 | 2.432 | **0.020*** |  |  |  |  |  |  |  |  |  |  |  |  |
| **P3b: amplitude** | | | |  |  |  |  |  |  |  |  |  |  |  |  |  |  |  |
| Intercept | 0.816 | 0.341 | | 50 | 2.393 | 0.021 |  |  |  |  |  |  |  |  |  |  |  |  |
| Baseline score | -0.011 | 0.094 | | 41 | -0.117 | 0.908 |  |  |  |  |  |  |  |  |  |  |  |  |
| Time | -0.527 | 0.143 | | 50 | -3.688 | **0.001**** |  |  |  |  |  |  |  |  |  |  |  |  |
| Treatment | -1.050 | 0.488 | | 21 | -2.151 | **0.043*** |  |  |  |  |  |  |  |  |  |  |  |  |
| Time*Treatment | 0.701 | 0.199 | | 50 | 3.514 | **0.001**** |  |  |  |  |  |  |  |  |  |  |  |  |
| **Cortical source localization of FPN & DMN within alpha frequencies**  **Early component P3a** | | | | | | |  |  |  |  |  |  |  |  |  |  |  |  |
| Intercept | 0.183 | 0.424 | | 39 | 0.431 | 0.669 |  |  |  |  |  |  |  |  |  |  |  |  |
| Baseline score | 0.063 | 0.117 | | 21 | 0.536 | 0.597 |  |  |  |  |  |  |  |  |  |  |  |  |
| Time | -0.094 | 0.236 | | 39 | -0.398 | 0.693 |  |  |  |  |  |  |  |  |  |  |  |  |
| Treatment | -0.394 | 0.616 | | 21 | -0.639 | 0.530 |  |  |  |  |  |  |  |  |  |  |  |  |
| Time*Treatment | 0.286 | 0.339 | | 39 | 0.842 | 0.405 |  |  |  |  |  |  |  |  |  |  |  |  |
| **Cortical source localization of FPN & DMN within alpha frequencies**  **Late component P3b** | | | | | | |  |  |  |  |  |  |  |  |  |  |  |  |
| Intercept | 0.566 | 0.360 | | 39 | 1.570 | 0.124 |  |  |  |  |  |  |  |  |  |  |  |  |
| Baseline score | 0.264 | 0.143 | | 19 | 1.843 | 0.081 |  |  |  |  |  |  |  |  |  |  |  |  |
| Time | -0.238 | 0.172 | | 39 | -1.382 | 0.175 |  |  |  |  |  |  |  |  |  |  |  |  |
| Treatment | -1.193 | 0.527 | | 21 | -2.265 | **0.034*** |  |  |  |  |  |  |  |  |  |  |  |  |
| Time*Treatment | 0.599 | 0.246 | | 39 | 2.432 | **0.02*** |  |  |  |  |  |  |  |  |  |  |  |  |
|  |  |  | |  |  |  |  |  |  |  |  |  |  |  |  |  |  |  |

**A.**


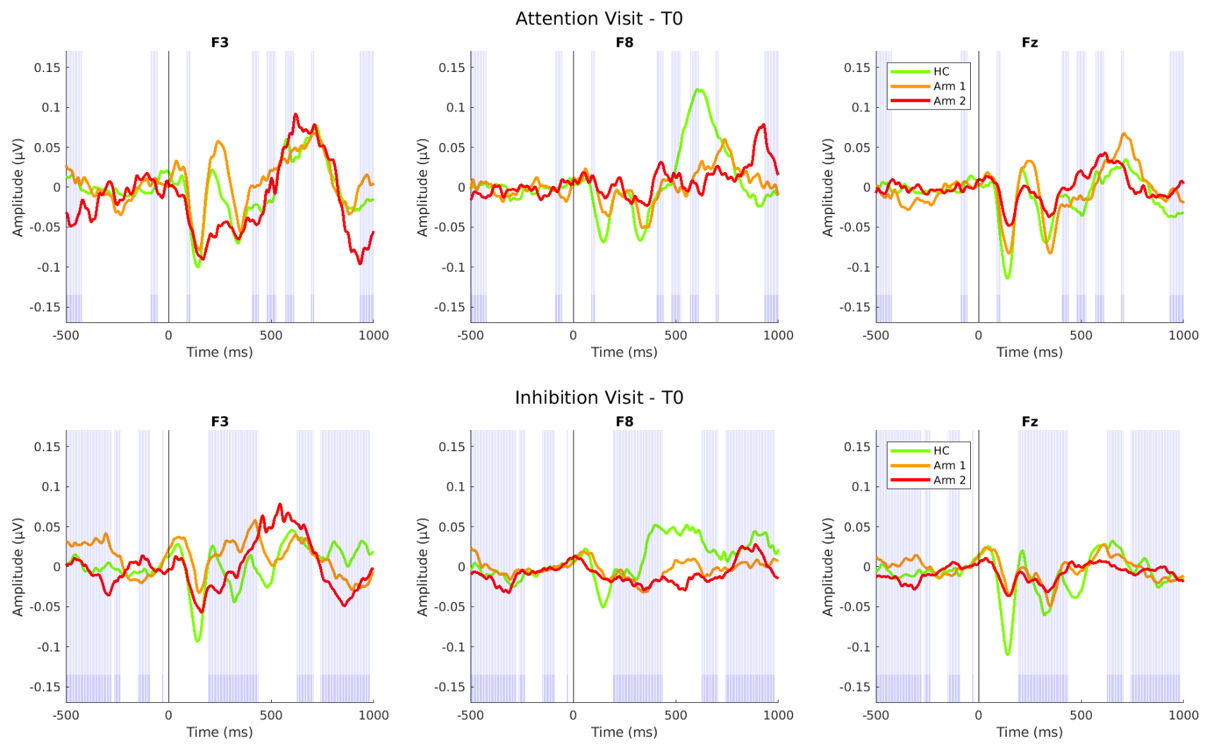


**B.**


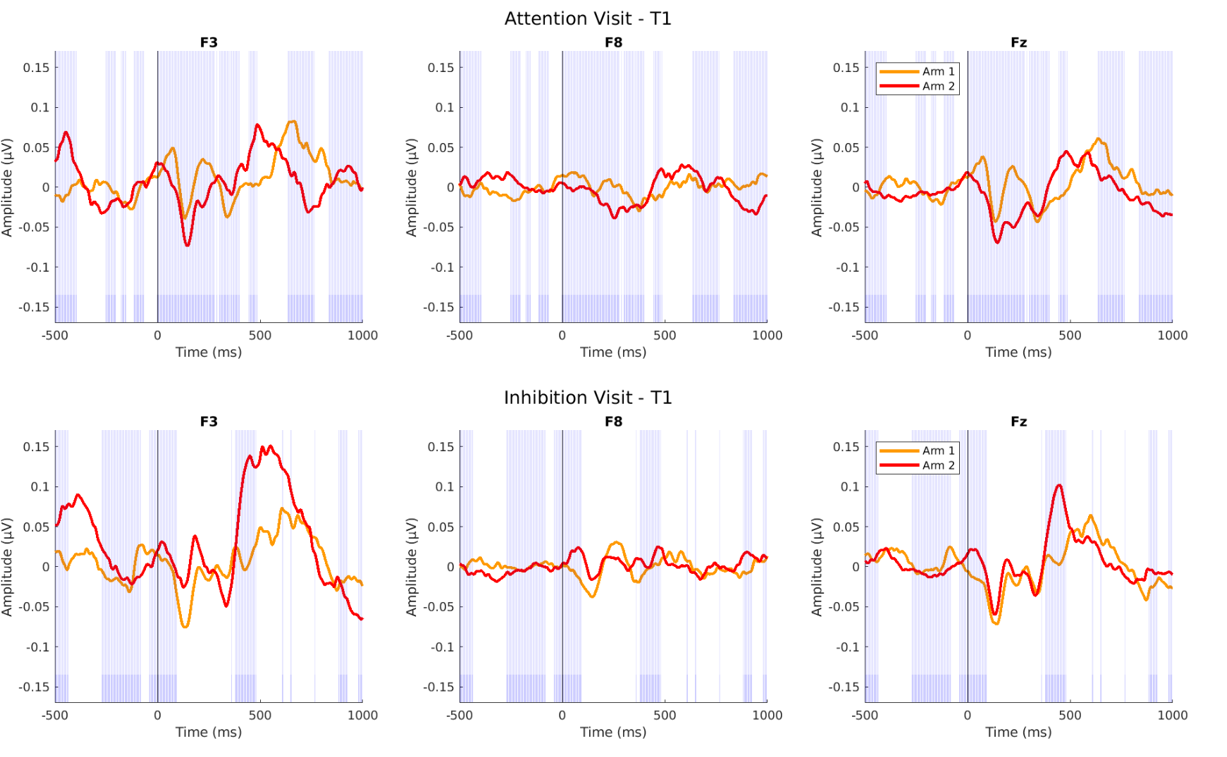


**C.**

**Supplementary Figure S2.** **Grand-Averaged ERP Waveforms Across Timepoints. Grand-averaged event-related potentials (ERPs)** recorded at frontal electrodes (F3, F8, Fz) during the **attention** and **inhibition** tasks for all groups and timepoints. At **baseline (t₀)**, data are shown for **Healthy Controls (HC; green), ADHD Arm 1 (tRNS + CT; orange),** and **ADHD Arm 2 (Sham + CT; red)**. Post-intervention (**t₁**) and follow-up (**t₂**) panels depict the two ADHD intervention arms only. The early **P3a (250–350 ms)** and late **P3b (400–600 ms)** intervals correspond to the major positive deflections across groups and sessions, confirming the time windows used for analysis
